# Meteoric Metal Chemistry in the Martian Atmosphere

**DOI:** 10.1002/2017JE005510

**Published:** 2018-03-06

**Authors:** J. M. C. Plane, J. D. Carrillo‐Sanchez, T. P. Mangan, M. M. J. Crismani, N. M. Schneider, A. Määttänen

**Affiliations:** ^1^ School of Chemistry University of Leeds Leeds UK; ^2^ Laboratory for Atmospheric and Space Physics (LASP) University of Colorado Boulder CO USA; ^3^ Laboratoire Atmosphères, Milieux Observations Spatiales (LATMOS) Guyancourt France

**Keywords:** cosmic dust, ablation, meteoric metals, Mars magnesium layer, Mars mesospheric clouds

## Abstract

Recent measurements by the Imaging Ultraviolet Spectrograph (IUVS) instrument on NASA's Mars Atmosphere and Volatile EvolutioN mission show that a persistent layer of Mg^+^ ions occurs around 90 km in the Martian atmosphere but that neutral Mg atoms are not detectable. These observations can be satisfactorily modeled with a global meteoric ablation rate of 0.06 t sol^−1^, out of a cosmic dust input of 2.7 ± 1.6 t sol^−1^. The absence of detectable Mg at 90 km requires that at least 50% of the ablating Mg atoms ionize through hyperthermal collisions with CO_2_ molecules. Dissociative recombination of MgO^+^.(CO_2_)_n_ cluster ions with electrons to produce MgCO_3_ directly, rather than MgO, also avoids a buildup of Mg to detectable levels. The meteoric injection rate of Mg, Fe, and other metals—constrained by the IUVS measurements—enables the production rate of metal carbonate molecules (principally MgCO_3_ and FeCO_3_) to be determined. These molecules have very large electric dipole moments (11.6 and 9.2 Debye, respectively) and thus form clusters with up to six H_2_O molecules at temperatures below 150 K. These clusters should then coagulate efficiently, building up metal carbonate‐rich ice particles which can act as nucleating particles for the formation of CO_2_‐ice clouds. Observable mesospheric clouds are predicted to occur between 65 and 80 km at temperatures below 95 K and above 85 km at temperatures about 5 K colder.

## Key Points

- A meteoric input function for Mars is generated by combining a cosmic dust input of ~3 tonnes sol^−1^ with a chemical ablation model
- MAVEN/IUVS observations of Mg^+^, and a small upper limit for Mg, suggest that around 50% of Mg atoms ionize directly after ablation
- MgCO_3_ and FeCO_3_ form stable H_2_O clusters which coagulate to metal‐rich ice particles, likely nuclei for clouds in the Martian mesosphere

## 1 Introduction

The ablation of cosmic dust in planetary atmospheres produces a continuous injection of metallic vapors such as Mg, Fe, and Na (Carrillo‐Sánchez et al., 2016; Plane, 2012). Although these metallic atoms and ions have been observed for decades in the terrestrial atmosphere (Plane et al., 2015), it was only recently that meteoric metals have been observed directly in another planetary atmosphere. The Imaging Ultraviolet Spectrograph (IUVS) (McClintock et al., 2014) on NASA's Mars Atmosphere and Volatile EvolutioN (MAVEN) mission has observed the dayglow emission at 280 nm from Mg^+^ ions. These ions occur as a layer in the Martian atmosphere between 70 and 130 km, peaking around 90 km with a peak density of ~350–400 cm^−3^ (Crismani et al., 2017).

These observations are somewhat in accord with atmospheric models of Mars magnesium chemistry (Molina‐Cuberos et al., 2003; Pesnell & Grebowsky, 2000; Whalley & Plane, 2010). For example, the most recent model (Whalley & Plane, 2010) predicted an Mg^+^ layer peak at 83 km with a significantly higher density of 1,800 cm^−3^; however, the absolute concentration is sensitive to the assumed injection rate of Mg from meteoric ablation, which in that study was a factor of 2–3 times higher than the IUVS observations imply (Crismani et al., 2017). More significantly, the model of Whalley and Plane predicted a much larger layer of neutral Mg atoms, peaking at 70 km with a density of 1 × 10^4^ cm^−3^. Scaling both layers so that the modeled Mg^+^ layer matches the IUVS observations would mean the Mg layer peaking at ~2,000 cm^−3^ and having a concentration at 90 km of ~300 cm^−3^. This altitude is where the IUVS instrument is most sensitive to measuring Mg resonance scattering at 285 nm, since at lower altitudes, the scattered solar continuum becomes dominant. Strikingly, the Mg detection upper limit at 90 km is only 130 cm^−3^ (Crismani et al., 2017), more than a factor of 2 smaller than the scaled model prediction. The disagreement is particularly surprising given that the Mg^+^ and Mg in the terrestrial upper atmosphere can be satisfactorily modeled (Langowski et al., 2015).

The problem of the “missing” Mg was identified by Crismani et al. (2017), in a study which included a preliminary examination of how the current understanding of meteoric ablation and the atmospheric chemistry of magnesium might need to be changed. The problem is quite challenging: If meteoric ablation injects the magnesium mostly as Mg atoms, or there is significant production of Mg from Mg^+^ or molecular species such as MgCO_3_, then the absence of a pronounced Mg layer cannot be explained in terms of the known chemistry of Mg. This is because the only known species in the Martian atmosphere with which Mg atoms can react are O_3_ and O_2_^+^ (as well as other more minor ions such as NO^+^); however, none of these reactants has a sufficiently high concentration to reduce Mg significantly (an O_3_ profile is shown in Figure S1 in the supporting information) (Crismani et al., 2017).

The first objective of the present study was therefore to investigate this problem in greater detail and show that a satisfactory reconciliation between the IUVS measurements and the model can be achieved. This also provides a more constrained estimate of the Mg meteoric injection rate. Our second objective was then to explore the fate of Mg and Fe (the two dominant meteoric metals) in the Martian mesosphere. The motivation for this is to understand whether meteoric material plays a significant role in providing ice nuclei for the formation of the CO_2_‐ice clouds that occur between 65 and 100 km. These clouds have been observed both from the surface of Mars (Smith et al., 1997) and from orbiting spacecraft (Clancy et al., 2007; Määttänen et al., 2010; Montmessin et al., 2007; Vincendon et al., 2011). The clouds occur more frequently close to aphelion, when the mesosphere is coldest, and also at low latitudes where the greater tidal activity produces cold pockets favoring the nucleation and growth of the ice particles, with gravity waves playing a secondary role (Spiga et al., 2012).

The CO_2_‐ice particles must form through heterogeneous nucleation because a sufficient degree of supersaturation (i.e., a cold enough temperature) is not reached in the Martian mesosphere for homogeneous nucleation to take place (Määttänen et al., 2010). Listowski et al. (2014) concluded that surface dust particles elevated into the mesosphere are insufficient to explain the observed daytime and nighttime clouds and suggested that an additional flux of meteoritic material is required. However, the source of the ice nuclei remains an unsolved problem. The nucleation of mesospheric H_2_O‐ice clouds in the terrestrial atmosphere (termed noctilucent or polar mesospheric clouds) is widely considered to occur on meteoric smoke particles (MSPs). MSPs form via the polymerization of molecules such as FeOH, Mg(OH)_2_, NaHCO_3_, and SiO_2_, which are the relatively long‐lived reservoir species produced by the oxidation of the metallic vapors injected by meteoric ablation (Plane et al., 2015). Recently, the nucleation and growth of CO_2_ ice on MSPs—represented by iron oxide and silica nanoparticles (radius *r* < 4 nm)—have been studied in the laboratory under conditions appropriate to Mars (Nachbar et al., 2016). For both types of particle, the contact parameter *m*, which governs nucleation in classical heterogeneous nucleation theory, was found to be only 0.78 ± 0.02, with no significant temperature dependence between 64 and 73 K. By applying this value for MSPs in the Martian mesosphere, the characteristic temperatures for the onset of CO_2_‐ice nucleation were shown to be 8–18 K below the CO_2_ frost point temperature. The result is that clouds can only be produced under exceptionally cold conditions, which does not seem to match their observed frequency of occurrence. One explanation for this would be that the MSPs which form in the Martian mesosphere are not in fact mineral grains but small H_2_O‐ice particles with a metal carbonate core. The reason for exploring this idea is that the value of *m* for CO_2_ condensing on H_2_O‐ice appears to be much larger: Glandorf et al. (2002) reported an average contact parameter of *m* = 0.95 on an ice substrate held between 130 and 140 K, although the lower limit of this temperature range is around 40 K higher than the temperatures at which the clouds occur.

## 2 Development of a Magnesium Model for Mars

The 1‐D model used for this study is an extension of our earlier model (Whalley & Plane, 2010). A simple eddy diffusion coefficient (*K*_zz_) profile (Figure S2) based on the previous literature is used: *K*_zz_ = 8 × 10^6^ cm^2^ s^−1^ below 90 km, optimized to give agreement between the modeled and IUVS‐measured peak of the Mg^+^ profile around 95 km (see section 3), and larger values (> 10^7^ cm^2^ s^−1^) above 100 km, which are consistent with the *K*_zz_ required to model measurements made by the Viking 1 and 2 spacecraft (Izakov, 1978) and values that have been used in other 1‐D models (Pesnell & Grebowsky, 2000; Rodrigo et al., 1990). The molecular diffusion coefficient profiles of Mg and Mg^+^ are also illustrated in Figure S2, which shows that the turbopause height would be around 116 km—consistent with measurements made by MAVEN's Neutral Gas Ion Mass Spectrometer during deep dip orbits, which extend periapse down to 130 km (Grebowsky et al., 2017). The turbopause and exobase are observed to move together in response to the warming of the lower atmosphere (Jakosky et al., 2017), and the Mg^+^ layer is expected to follow suit. Therefore, altitudes considered here are relevent for the stated observing conditions and are not intended to represent the Mg^+^ layer generally.

The Mars Climate Database v.5.2 (http://www-mars.lmd.jussieu.fr/mcd_python/) (Forget et al., 1999) is used to provide vertical profiles of temperature, pressure, and the mixing ratios of CO_2_, O_2_, O_3_,O, O_2_, and CO (Figure S1). The electron density profile is also taken from the Climate Database, and electrons are assumed to be balanced by O_2_^+^ ions (González‐Galindo et al., 2013). Daytime low‐latitude conditions are used (local noon, latitude = 0°, solar longitude *L*_*s*_ = 85°).

**Figure 1:**
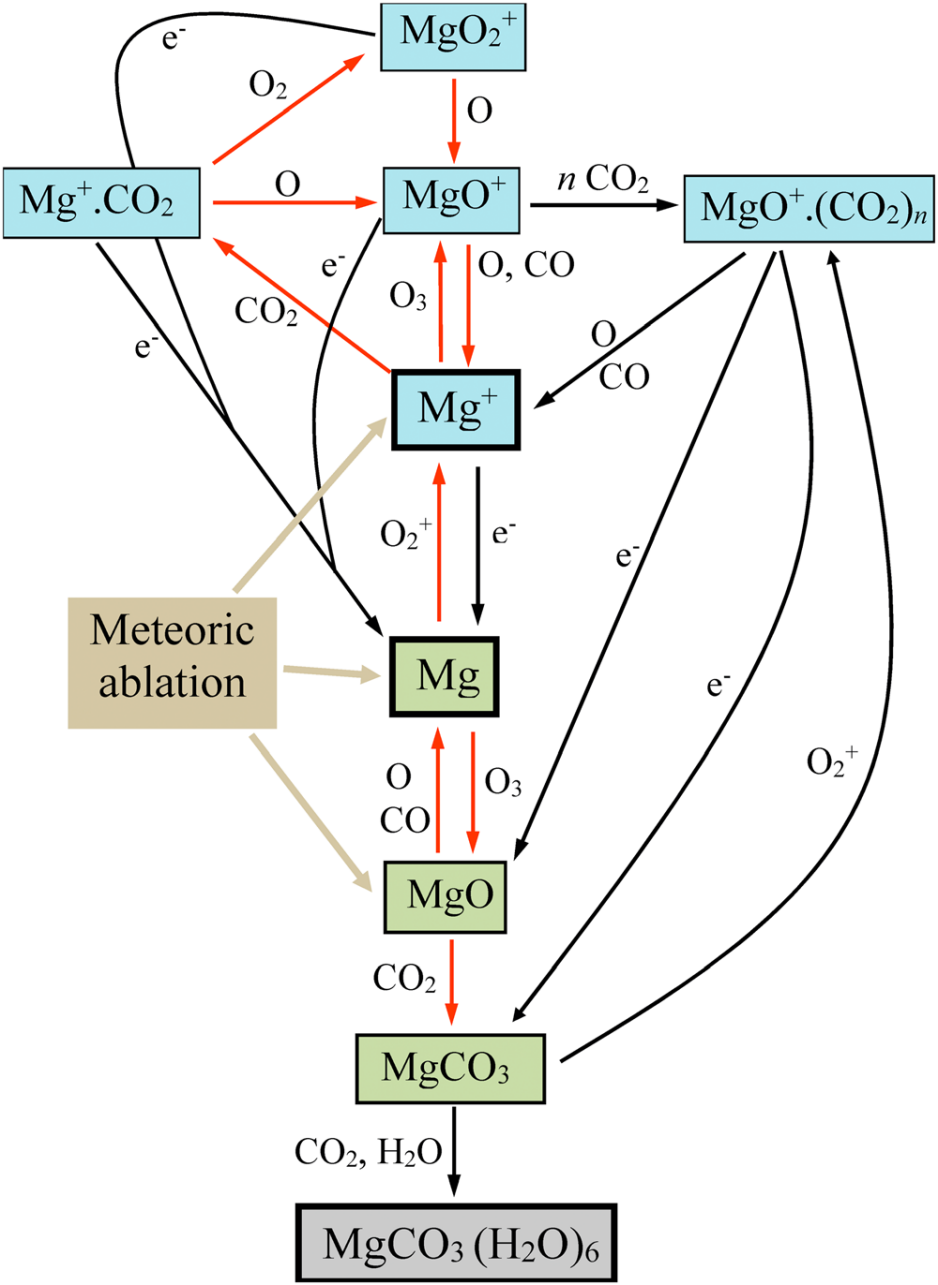
A schematic diagram of the neutral and ion‐molecule chemistry of magnesium in the Martian upper atmosphere. Ionized and neutral compounds are shown in blue and green boxes, respectively. The first building block of metal‐rich ice particles is shown in gray. Reactions with measured rate coefficients (see Table 1) are indicated with red arrows.

Table 1 lists the reactions and rate coefficients for the reaction scheme used in the 1‐D model, which is illustrated in Figure 1. Many of these rate coefficients have now been measured over the temperature and pressure ranges needed to extrapolate with reasonable confidence to the conditions on Mars (see the footnotes to the table). Note that the charge transfer reaction of Mg with ambient O_2_^+^ ions (and less abundant NO^+^ and CO_2_^+^ ions) is much more important than photoionization above 70 km as the source of Mg^+^, which is also the case for the other major metallic ion Fe^+^ (Figure S3) (Whalley & Plane, 2010).

**Table 1:** Rate Coefficients for Important Reactions of Mg and Mg^+^ in the Mars Atmosphere

	Neutral reactions	
R1	Mg + O_3_ → MgO + O_2_	2.3 × 10^−10^ exp(−139 K/*T*) a
R2	MgO + O → Mg + O_2_	5.8 × 10^−10^ (*T*/200 K)^1/6^ b
R3	MgO + CO → Mg + CO_2_	1.5 × 10^−11^ (*T*/200 K)^−0.87^ b
R4	MgO + CO_2_ (+CO_2_) → MgCO_3_ → MgCO_3_.(H_2_O)_6_	5.9 × 10^−29^ (*T*/200 K)^−0.86^ c ^,^ ♣
	Ion‐molecule reactions	
R5	Mg + O_2_^+^ → Mg^+^ + O_2_	1.2 × 10^−9^ d
R6	Mg + NO^+^ → Mg^+^ + NO	8.2 × 10^−10^ d
R7	MgCO_3_ + O_2_^+^ → MgO^+^.CO_2_ + O_2_	2 × 10^–9^ e
R8	Mg^+^ + O_3_ → MgO^+^ + O_2_	1.2 × 10^–9^ f
R9	MgO^+^ + O → Mg^+^ + O_2_	5.9 × 10^−10^ b
R10	MgO^+^ + CO → Mg^+^ + CO	3.2 × 10^–10^ h
R11	MgO^+^ + CO_2_ (+CO_2_) → MgO^+^.CO_2_ → MgO^+^.(CO_2_)_*n*_	2.8 × 10^−26^ (*T*/200 K)^−3.12^ i ^,^ ♠
R12	MgO^+^.(CO_2_)_*n*_ + O → Mg^+^ + O_2_ + *n*CO_2_	5.9 × 10^−10^ c
R13	MgO^+^.(CO_2_)_*n*_ + CO → Mg^+^ + (*n* + 1)CO_2_	3.2 × 10^–10^ j
R14	Mg^+^ + CO_2_ (+CO_2_) → Mg^+^.CO_2_	5.6 × 10^−29^ (*T*/200 K)^−1.59^ g ^,^ ♠
R15	Mg^+^.CO_2_ + O → MgO^+^ + CO_2_	6.5 × 10^−10^ b
R16	Mg^+^.CO_2_ + O_2_ → MgO_2_^+^ + CO_2_	2.2 × 10^−11^ g
R17	MgO_2_^+^ + O → MgO^+^ + O_2_	6.5 × 10^−10^ b
R18	Mg^+^.CO_2_ + CO_2_ (+CO_2_) → Mg^+^.(CO_2_)_2_ → MgO^+^.(CO_2_)	1.4 × 10^−27^ (*T*/200 K)^−5.08^ g ^,^ ♠
R19	Mg.X^+^ + e^−^ → Mg + X (X = O, O_2_, CO_2_)	2.4 × 10^−7^ (*T*/200 K)^−0.5^ k
R20	MgO^+^.(CO_2_)_*n*_ + e^−^ → MgCO_3_ + (*n* − 1)CO_2_ → MgO + *n*CO_2_	*β* × 2.4 × 10^−7^ (*T*/200 K)^−0.5 ^ k ^,^ l (1 − *β*) × 2.4 × 10^−7^ (*T*/200 K)^−0.5^
R21	Mg^+^.(CO_2_)_*n*_ + e^−^ → Mg + *n*CO_2_	2.4 × 10^−7^ (*T*/200 K)^−0.5^ k
R22	Mg^+^ + e^−^ → Mg + *hv*	3.3 × 10^−12^ (*T*/200 K)^−0.64^ m

♣ Recombination reaction where the rate coefficient measured in N_2_ has been multiplied by a factor of 2 to correct for CO_2_ as third body.

♠ Recombination reaction where the rate coefficient measured in He has been multiplied by a factor of 8 to correct for CO_2_ as third body.

a Plane and Helmer (1995).

b Plane and Whalley (2012).

c Rollason and Plane (2001).

d Rutherford et al. (1971).

e Estimate taking account of the dipole moment of MgCO_3_.

f Whalley et al. (2011).

g Whalley and Plane (2010), R12 set to lower limit of extrapolation from experimental T range.

h Rowe et al. (1981).

i Calculated using Rice‐Ramsperger‐Kassel‐Markus theory (see text).

j Assumed to have the same rate constant as the MgO^+^ reaction.

k Estimate base on review of dissociative electron recombination (Florescu‐Mitchell & Mitchell, 2006).

l *β* is the product branching ratio; values of 1 and 0.75, respectively, are used in the standard model.

m Badnell (2006).

### 2.1 Meteoric Ablation

**Figure 2:**
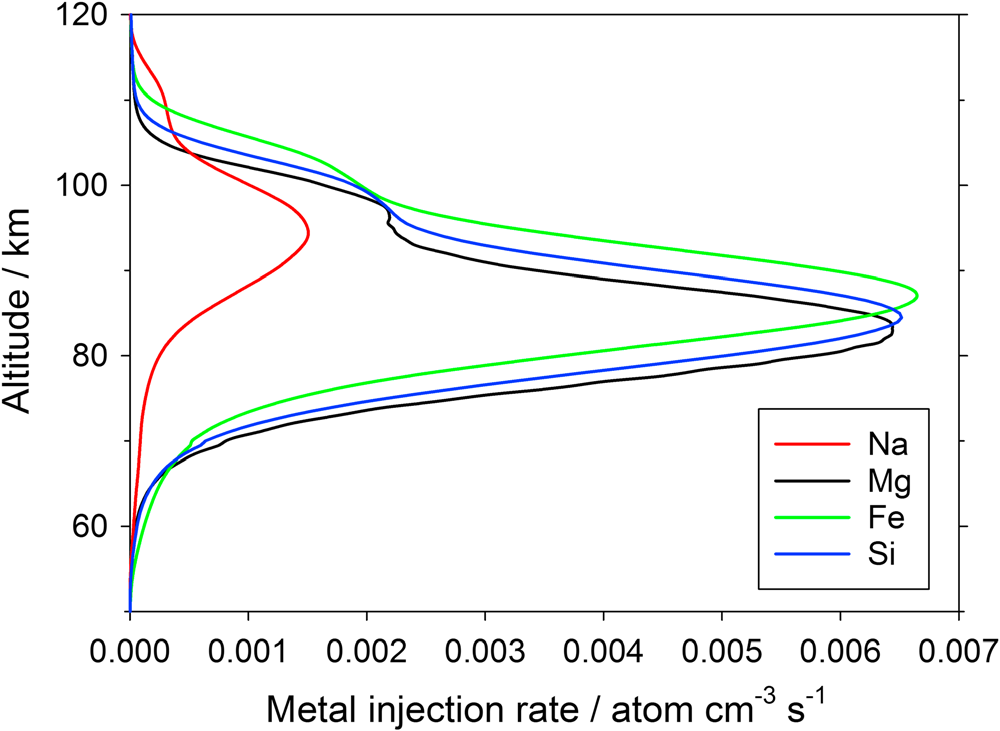
Injection rate of Na, Mg, Fe, and Si as a function of altitude, from the ablation of cosmic dust entering Mars atmosphere.

The profile of the Mg injection rate from meteoric ablation is shown in Figure 2. This was calculated using the Leeds Chemical Ablation Model (CABMOD) (Vondrak et al., 2008), which estimates the rates of ablation of individual elements from a meteoroid of specified mass, density, entry velocity, and entry angle. The following processes are included: sputtering by inelastic collisions with air molecules, which can be important before the meteoroid melts; after melting, thermodynamic equilibrium of the molten phase and the vapor around the particle; and the evaporation rate of each element (atom or oxide) calculated assuming Langmuir evaporation. We have recently used this model to determine the absolute fluxes of cosmic dust particles entering the terrestrial atmosphere from three sources: short‐period Jupiter Family Comets, Long Period Comets, and Asteroids (Carrillo‐Sánchez et al., 2016), where the size and velocity distributions of dust from each source are determined from an astronomical model (Nesvorný et al., 2010, 2011). This model, constrained by terrestrial observations, is now applied to Mars.

Integrating over the mass and velocity distributions from the different dust sources yields the absolute injection rates of different elements, illustrated in Figure 2 for Mg, Fe, Na, and Si. The peak height and absolute injection rate of Mg are in good agreement with the previous Mars models of Molina‐Cuberos et al. (2003) and Whalley and Plane (2010). The peak injection rate is around 83 km, which is ~10 km lower than in the Earth's atmosphere (Carrillo‐Sánchez et al., 2016). This is because the mean orbital velocity of Mars is 24.1 km s^−1^ and its escape velocity is 5.0 km s^−1^, compared with 29.8 and 11.2 km s^−1^ for Earth. Extrapolating globally, the ablation input of Mg species would be 0.06 t sol^−1^, out of a total dust input at Mars of 2.7 ± 1.6 t sol^−1^. The uncertainty in the global input rate is estimated using the Monte Carlo procedure we used previously to fit the contribution from the three cosmic dust sources entering the terrestrial atmosphere (Carrillo‐Sánchez et al., 2016); this uncertainty is then (arbitrarily) doubled because the Mars input is unconstrained apart from the IUVS measurements. The total input rate agrees well with our recent estimate of 2–3 t sol^−1^ (Crismani et al., 2017).

We now consider the fraction of ablating magnesium that forms neutral atomic Mg. CABMOD predicts that only 16% of the Mg in the incoming particles ablates, because MgO in the silicate melt is relatively refractory and so most of the Mg ablates from particles traveling faster than 8 km s^−1^. The kinetic energy of the freshly ablated Mg is then above the ionization energy of Mg (7.64 eV), so that Mg^+^ could be produced through hyperthermal collisions. There are two further possibilities. First, these high‐energy collisions can also produce Mg in its low‐lying metastable Mg(^3^P) state, which is 2.7 eV above the (^1^S) ground state. The ^3^P_1_ spin‐orbit state decays radiatively in 4 ms, and the ^3^P_2_ and ^3^P_0_ multiplets are dark states. Since the statistical ratio of these dark states to the ^3^P_1_ state is 2.8:1, at least 73% of the Mg(^3^P) will survive long enough to undergo collisions with CO_2_ molecules, which produces MgO efficiently (Cox & Dagdigian, 1984; Taieb & Broida, 1976). Second, collisions of ground‐state Mg(^1^S) with CO_2_ can be sufficiently energetic to produce MgO + CO directly, since this reaction is endothermic by only 2.8 eV (Plane & Whalley, 2012).

**Figure 3:**
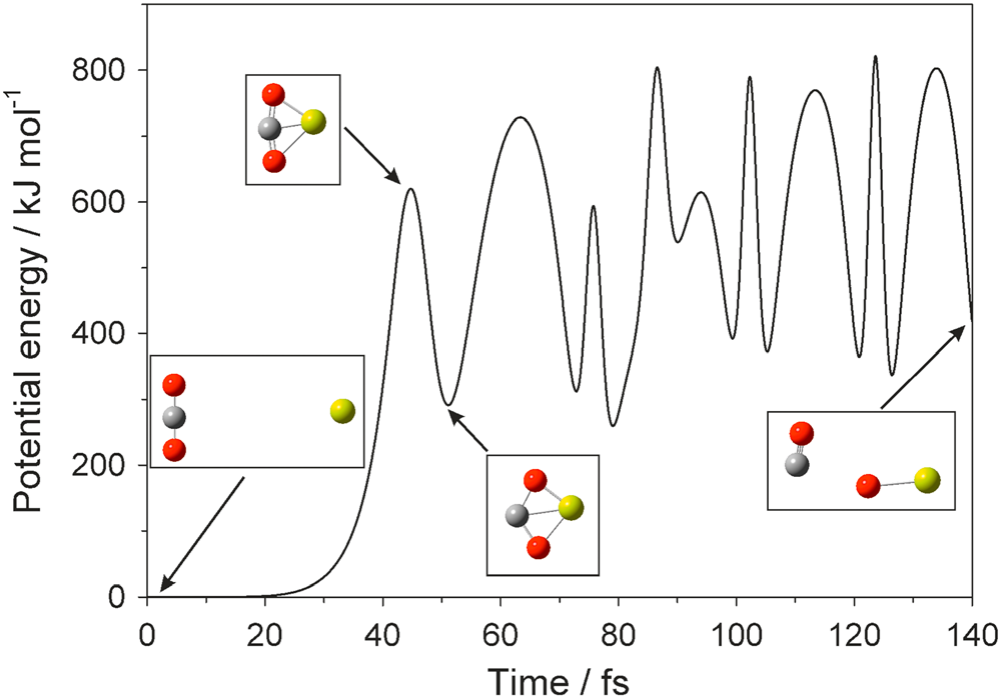
Trajectory calculation of Mg colliding with CO_2_ at a relative velocity of 10.8 km s^−1^ and producing MgO + CO. The change in potential energy as a function of time is shown, along with the molecular geometries at four points along the trajectory (Mg: yellow; oxygen: red; carbon: gray). Level of theory: b3lyp/6‐311+g(2d,p). An animation of the trajectory is included in movie S2.

We explored these options by simulating hyperthermal collisions using quantum chemistry dynamics calculations. The AtomCentered Density Matrix Propagation (ADMP) method (Schlegel et al., 2002) was employed, as implemented in the Gaussian 09 suite of programs (Frisch et al., 2009). The electronic structure model used for the ADMP calculations was B3LYP density functional theory with the 6‐311+g(2d,p) basis set. The ADMP trajectory steps were 0.1 fs long and used fully converged self‐consistent field results. Figure 3 is an example of trajectory where Mg reacts with CO_2_ to produce MgO and CO. Note the large amount of vibrational excitation in the products. An animation of the trajectory is available in Movie S2 in the supporting information. An example of a trajectory which produces excited Mg(^3^P), CO, and O(^3^P), thus conserving overall spin since Mg + CO_2_ begins on a surface with singlet spin, is also shown in Figure S4 and Movie S1.

### 2.2 Neutralization of Mg‐Containing Ions

As shown in Figure 1, a variety of small molecular ions—Mg^+^.CO_2_, MgO^+^, and MgO_2_^+^—form from Mg^+^. If these ions then undergo dissociative recombination (DR) with electrons, Mg atoms will be the most likely product. In order to avoid the resulting buildup of neutral Mg to levels observable by the IUVS instrument at 90 km, we considered the role of clustering in a CO_2_ atmosphere. First, the clustering of CO_2_ with MgO^+^ (the reaction numbering follows that in Table 1):
(R11)${\mathrm{MgO}}^{+}+{\mathrm{CO}}_{2}\;\left(+{\mathrm{CO}}_{2}\right)\to {\mathrm{MgO}}^{+}.{\mathrm{CO}}_{2}\;{{\text{or MgCO}}_{3}}^{+}$


**Figure 4:**
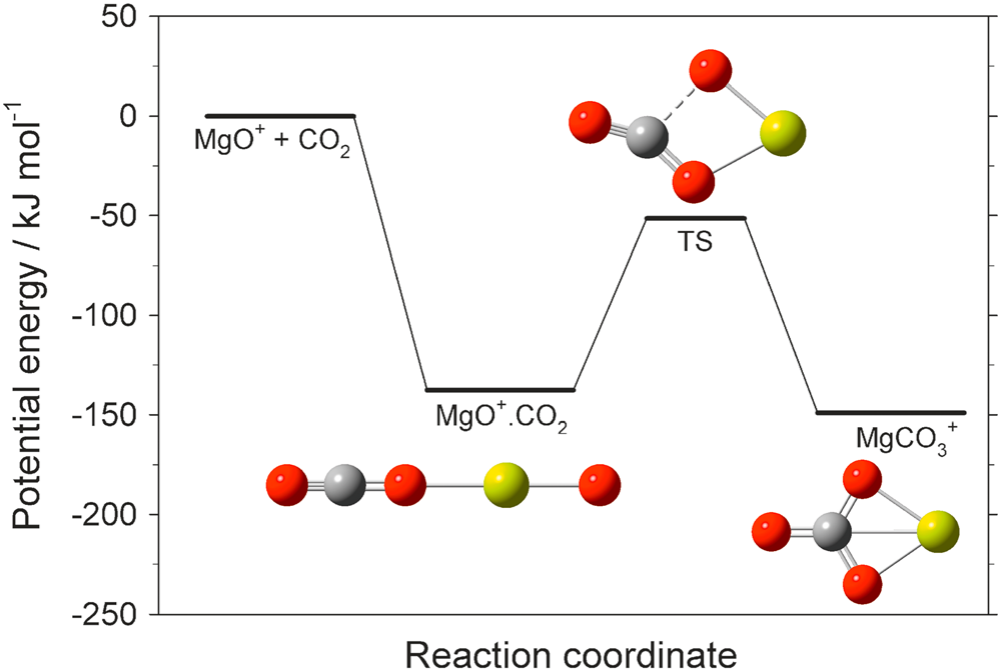
Potential energy surface for the addition of CO_2_ to the MgO^+^ ion, calculated at the CBS‐QB3 level of theory (Mg: yellow; O: red; C: gray).

The rate coefficient for this reaction was calculated using electronic structure theory to determine the reaction enthalpy (149 kJ mol^−1^) at the complete basis set (CBS‐QB3) level of theory from Petersson and coworkers (Montgomery et al., 2000). The molecular parameters (rotational constants and vibrational frequencies) required to apply Rice‐Ramsperger‐Kassel‐Markus theory (Whalley et al., 2011) are listed in Table S1 in the supporting information. Figure 4 is a potential energy surface for the reaction. Although both products are accessible (the transition state between them is below the entrance channel of the reaction), the density of states of the loosely‐bound MgO^+^.CO_2_ cluster is much higher than the MgCO_3_^+^ ion, and so this is the preferred product. The calculated rate coefficient is listed in Table 1. Once formed, further CO_2_ molecules are likely to cluster to produce MgO^+^.(CO_2_)_*n*_, which can then undergo DR:
(R20)$\begin{array}{l} {\mathrm{MgO}}^{+}.{\left({\mathrm{CO}}_{2}\right)}_{n}+e^{-}\to {\text{MgCO}}_{3}+\left(n-1\right){\mathrm{CO}}_{2}\;\beta \\ \;\to \mathrm{MgO}+{\left({\mathrm{CO}}_{2}\right)}_{n}\;1-\beta \; \end{array}$


where *β* is the reaction branching ratio to form MgCO_3_, thus potentially avoiding formation of Mg since MgO is likely to be rapidly reduced to Mg by reaction with O and CO (Figure 1).

Second, the clustering of CO_2_ with Mg^+^.CO_2_, which we have measured to be a fast association reaction (Whalley et al., 2011):
(R18)${\mathrm{Mg}}^{+}.{\mathrm{CO}}_{2}+{\mathrm{CO}}_{2}+\left(+{\mathrm{CO}}_{2}\right)\to {\mathrm{Mg}}^{+}.{\left({\mathrm{CO}}_{2}\right)}_{2}$


Further clustering with CO_2_ is likely, followed at some point by ligand‐switching with O:

$${\mathrm{Mg}}^{+}.{\left({\mathrm{CO}}_{2}\right)}_{n}+O\to {\mathrm{MgO}}^{+}.{\left({\mathrm{CO}}_{2}\right)}_{n-1}+{\mathrm{CO}}_{2}$$

This reaction will be more likely than DR with electrons because, although the rate coefficient for this reaction—which is around 6 × 10^−10^ cm^3^ molecule^−1^ s^−1^ (Whalley et al., 2011)—is ~500 times slower than the DR reaction, the ratio of [O]/[e^−^] exceeds 10^5^ at altitudes below 110 km. Thus, in the model, we assume that R18 leads to formation of MgO^+^.(CO_2_)_*n*_.

In the 1‐D model the sink for MgCO_3_ is assumed to be clustering with other meteoric compounds. This is treated here in two ways. The simpler approach is to include a dimerization reaction of MgCO_3_ with itself, where (MgCO_3_)_2_ is treated as a permanent sink (Plane, 2004). The dimerization rate coefficient is assigned to be 4.5 × 10^−9^ cm^3^ molecule^−1^ s^−1^, which is 5 times larger than the dipole‐dipole capture rate of MgCO_3_ + MgCO_3_. This reflects the fact that an MgCO_3_ molecule could polymerize with other metal carbonates as well as MgCO_3_ and also react with SiO_2_. The ratio of all ablating meteoric metals to Mg is ~4:1 (section 2.1), which we arbitrarily increase by 25% to 5:1 to allow for reaction with larger clusters. The second approach is to use the ice particle surface area calculated in section 2.3 to provide a heterogeneous sink for MgCO_3_, with an uptake coefficient of unity. Both approaches yield very similar results for the modeled Mg and Mg^+^ layers.

Figure S5 illustrates the potential energy surface for the FeO^+^ + CO_2_ reaction, analogous to R18. The surface is similar to that for MgO^+^ + CO_2_, although the barrier between FeO^+^.CO_2_ and FeCO_3_^+^ is now higher than the reactant entrance channel, and so FeO^+^.CO_2_ should be the exclusive product. The molecular properties of the surface are listed in Table S2. Application of Rice‐Ramsperger‐Kassel‐Markus theory (see section 2.2) indicates that this reaction should be slightly slower than R1: *k*(FeO^+^ + CO_2_) = 1.1 × 10^−26^ (*T*/200 K)^−3.11^ cm^6^ molecule^−2^ s^−1^. It is therefore likely that meteor‐ablated Fe will follow a similar path to FeCO_3_ as that illustrated in Figure 1 for Mg.

### 2.3 Formation of Neutral Clusters of MgCO_3_ and FeCO_3_

Both MgCO_3_ and FeCO_3_ have extremely large dipole moments: 11.6 and 9.2 Debye, respectively, at the B3LYP/6‐311+g(2d,p) level of theory. We now examine the clustering of CO_2_ and H_2_O to these polar molecules. The Gibbs free energies of the clusters were calculated up to sizes relevant for the temperature and pressure conditions of the Martian mesosphere (5 CO_2_ ligands and 10 H_2_O ligands). For computational efficiency, calculations were performed at the B3LYP/6‐311+g(2d,p) level, which provides a compromise between tolerable accuracy (which we have previously benchmarked against higher levels of theory; Plane, 2011) and the demanding computational resources required to perform calculations (including vibrational frequencies) on systems with up to 35 atoms.

The geometries of MgCO_3_ and FeCO_3_ and their clusters were first optimized. As we have shown previously (Rollason & Plane, 2000, 2001), these molecules have planar, kite‐shaped structures with C_2v_ symmetry. Electronically, MgCO_3_ has a singlet spin state, that is, the molecule has no unpaired electrons, whereas FeCO_3_ is in a quintet spin state with four unpaired electrons. After optimizing the molecular geometry of each species, the vibrational frequencies were calculated in order to make zero‐point energy corrections to the cluster binding energies. The vibrational frequencies and rotational constants were then used to compute the binding entropies, and hence, the Gibbs free energy changes for cluster formation. The molecular parameters for MgCO_3_‐(CO_2_)_1–4_, FeCO_3_(CO_2_)_1–3_, MgCO_3_‐(H_2_O)_1–10_, and FeCO_3_‐(H_2_O)_1–10_ are listed in Tables S3, S4, S5, and S6, respectively. It should be noted that some of the clusters have vibrational modes with frequencies less than 100 cm^−1^, which really correspond to hindered internal rotations of the CO_2_ or H_2_O molecules against the metal carbonate. In fact, treating these low‐frequency vibrations in the limit as free rotors over the pertinent temperature range of 80–150 K (using the method of Benson, 1968) produces little difference in the calculated values of Δ*G*_*n* − 1,*n*_, the Gibbs free energy change for the addition of a CO_2_ or H_2_O molecule to a cluster of MgCO_3_ and FeCO_3_ with *n* − 1 ligands. Hence, the rotational and vibrational partition functions were calculated assuming that the clusters are rigid rotors and that all vibrations are harmonic. The Δ*G*_*n* − 1,*n*_ values are listed in Tables S7–S10, over a range of temperatures from 80 to 150 K.

**Figure 5:**
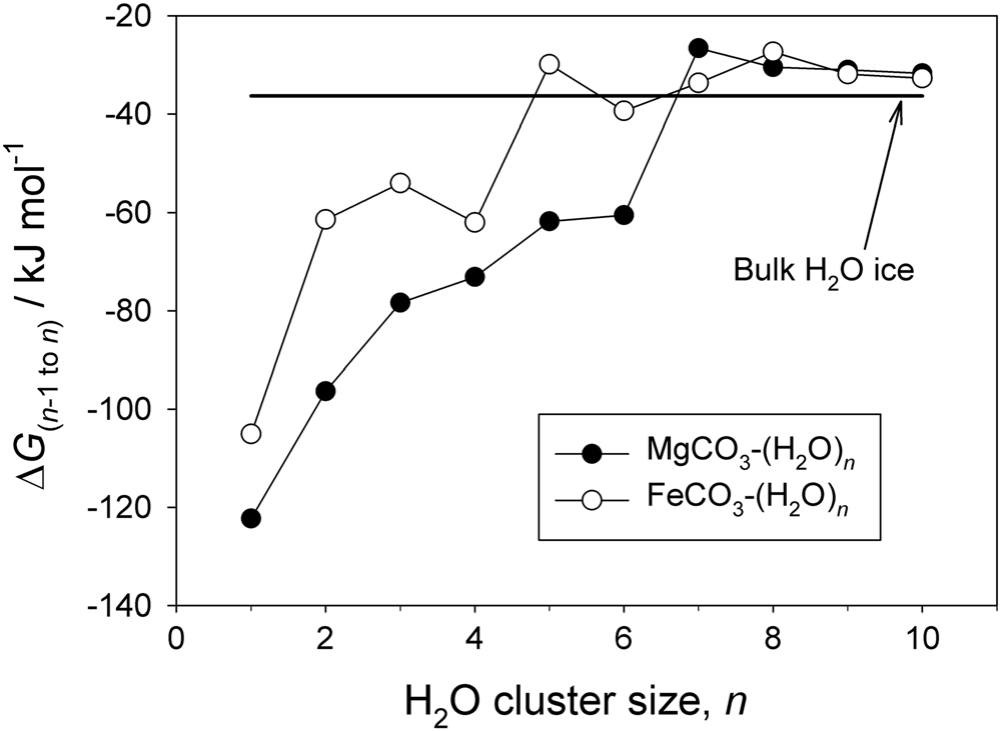
Change in Gibbs free energy (Δ*G*) at 100 K for the formation of the *n*th H_2_O cluster by addition of a single H_2_O molecule to the (*n* − 1)th cluster, for MgCO_3_ and FeCO_3_.

Figure 5 illustrates the dependence of Δ*G*_*n* − 1,*n*_ on *n* for the addition of H_2_O to MgCO_3_ and FeCO_3_ at 100 K. This shows that the free energy change is strongly favorable (i.e., negative) for the formation of the first few clusters (*n* = 6 for MgCO_3_, *n* = 4 for FeCO_3_), and then Δ*G* approaches that for sublimation of an H_2_O to a bulk ice surface, determined from the vapor pressure above H_2_O‐ice at 100 K (Murphy & Koop, 2005, equation (7)). In the Martian mesosphere between 70 and 90 km, MgCO_3_‐(CO_2_)_*n*_ clusters up to *n* = 3 are stable at temperatures below 150 K. Given that CO_2_ is the major atmospheric constituent, MgCO_3_‐(CO_2_)_3_ will form very rapidly, followed by ligand exchange with H_2_O. For example, for the ligand‐switching reaction

$${\text{MgCO}}_{3}-{\left({\mathrm{CO}}_{2}\right)}_{3}+H_{2}O\to {\text{MgCO}}_{3}-\left({\mathrm{CO}}_{2}\right)-H_{2}O+{\mathrm{CO}}_{2}$$

Δ*G* ranges from −58.7 to −58.3 kJ mol^−1^ between 80 and 150 K, and so switching is overwhelmingly favored despite the H_2_O mixing ratio being only ~0.7 parts per million between 65 and 90 km (http://www-mars.lmd.jussieu.fr/mcd_python/) (Forget et al., 1999). This means that the three CO_2_ molecules that originally clustered with the MgCO_3_ will be displaced by H_2_O, and then further addition of H_2_O will occur up to MgCO_3_‐(H_2_O)_6_, which is stable at all temperatures below 150 K between 60 and 90 km.

**Figure 6:**
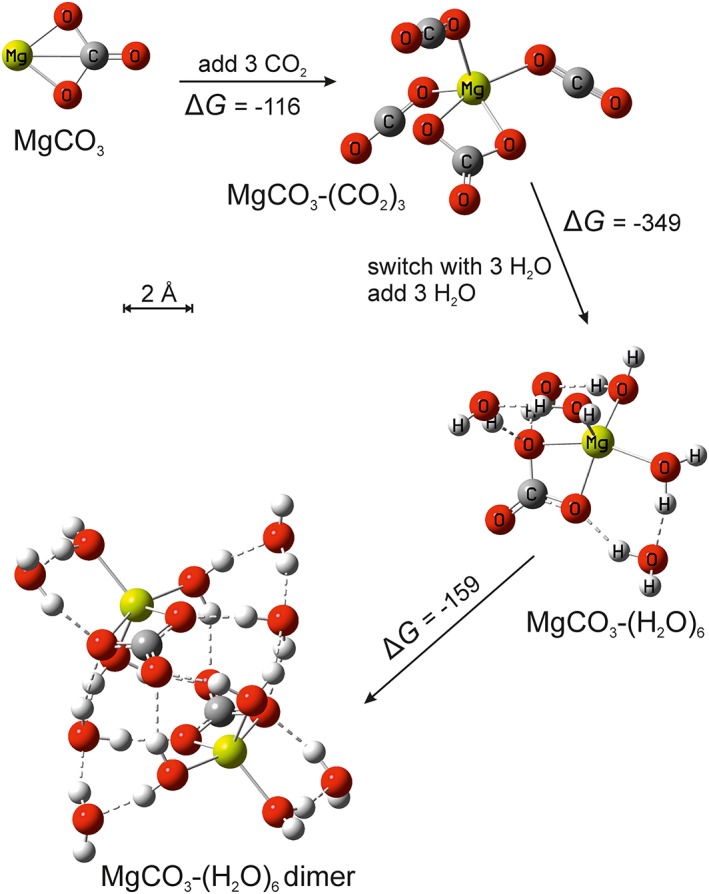
Route from MgCO_3_ to the stable ice nanoparticle. Initially up to four CO_2_ molecules can bind to the MgCO_3_, but these switch with the stronger H_2_O ligand to form MgCO_3_(H_2_O)_6_. These clusters can then coagulate with favorable free energies (Δ*G*, in kJ mol^−1^).

Figure 6 illustrates this sequence from MgCO_3_ to MgCO_3_‐(H_2_O)_6_. The MgCO_3_‐(H_2_O)_6_ particles will form relatively rapidly compared to the coagulation rate of metal‐containing compounds, and so MgCO_3_‐(H_2_O)_6_ is likely to be the building block for larger particle. The first step in coagulation will be production of the MgCO_3_‐(H_2_O)_6_ dimer, a reaction which is highly exothermic so that Δ*G* ranges from −174 to −157 kJ mol^−1^ between 80 and 150 K (see Figure 6). Further coagulation should be equally strongly favored. FeCO_3_ has a smaller dipole moment than MgCO_3_ (see above) and so forms less stable clusters. FeCO_3_‐(CO_2_)_*n*_ clusters up to *n* = 1 are stable below 150 K and *n* = 2 below 120 K. Ligand‐switching with H_2_O and further addition of H_2_O will then build up FeCO_3_‐(H_2_O)_6_ clusters which are stable below 130 K. Other metal carbonates (e.g., NaCO_3_ with a dipole moment of 8.7 Debye at the B3LYP/6‐311+g(2d,p) level) will also produce H_2_O clusters in the same way.

The only meteoric ablation product that should not form H_2_O clusters is Si. This element will initially form SiO_2_, which will then hydrolyze to silicic acid (Si(OH)_4_) (Plane et al., 2016). This symmetric molecule with the four OH groups arranged in a tetrahedron around the Si atom does not have a dipole moment, so that H_2_O can only hydrogen bond with a bond energy of 24 kJ mol^−1^ (at the B3LYP/6‐311+g(2d,p) level), which is too small to produce a sufficiently favorable free energy. We therefore assume that—under steady state background conditions—the rate of injection of metal carbonate‐H_2_O clusters is equal to the rate of ablation of all metals apart from Si. In fact, Mg and Fe make up 89% of the non‐Si injection rate between 80 and 90 km.

The subsequent growth of particles via coagulation is then treated using a semi‐implicit, volume‐conserving sectional model (Saunders & Plane, 2006). Growth takes place through a number of discrete size bins where the first bin size (*r*_1_ = 0.46 nm) corresponds to the radius of a MgCO_3_‐(H_2_O)_6_ cluster (Figure 6), with an effective particle density of 780 kg m^−3^. The size (*r*_*i*_) of successive bins is scaled geometrically (i.e., *r*_*i* + 1_ = *f* ^1/3^ *r*_*i*_, where *f* = 2.0), so that the radius of the largest of the 40 bins in the model is *r*_40_ = 3.8 μm.

Extrapolating from the large negative Δ*G* for the dimerization of MgCO_3_‐(H_2_O)_6_ clusters (see above), collisions between particles in all size bins are assumed to proceed spontaneously (i.e., without a thermodynamic barrier). Growth is then dominated by Brownian diffusion and coagulation. Collisions between pairs of particles are assumed to result in coalescence, where the spherical morphology and relatively open structure (particle density = 780 kg m^−3^) are maintained. Note that the effective density of the MgCO_3_‐(H_2_O)_6_ dimer in Figure 6 is only 595 kg m^−3^. However, as discussed below (section 3.2), residual gas‐phase H_2_O may condense onto these particles, so that their densities would increase toward that of low density amorphous H_2_O ice (940 kg m^−3^; Jenniskens & Blake, 1994). Following Fuchs (1964), the coagulation rate coefficients of the smaller particles are calculated using an expression for the free molecular regime (i.e., Knudsen number, *K*_*n*_ ≫ 1), which is interpolated into the transition regime for larger particles. Gravitational sedimentation of particles in each size bin is calculated using Stokes's law, modified for the slip‐flow regime (Jacobson, 2005).

## 3 Results and Discussion

### 3.1 The Mg^+^ and Mg Layers

**Figure 7:**
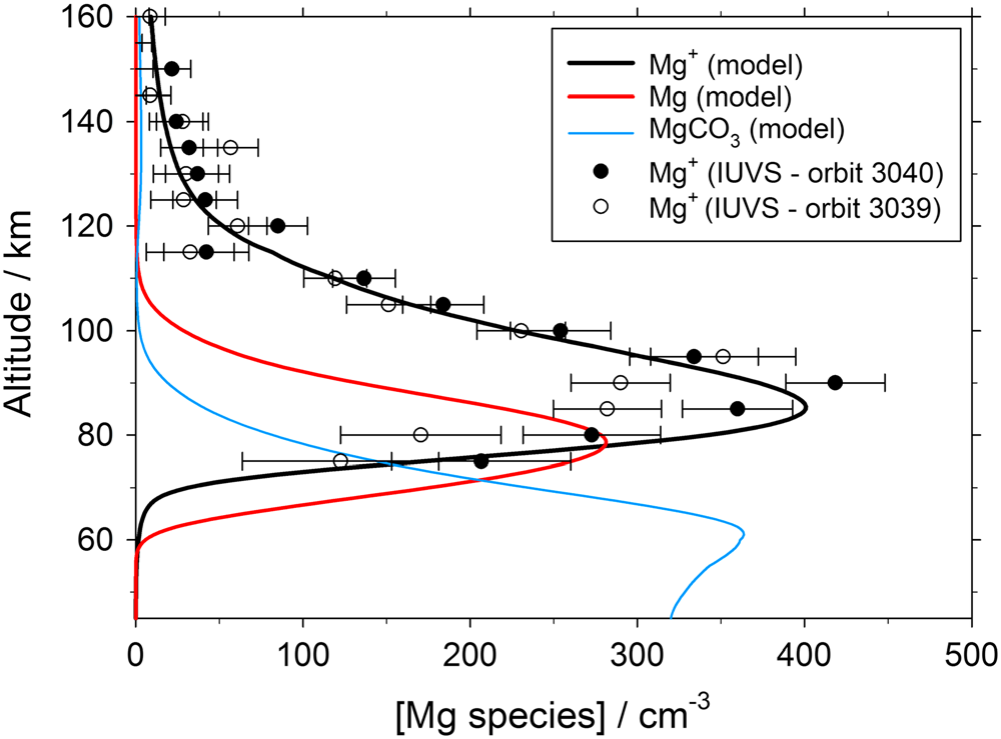
Vertical profiles of Mg^+^, Mg, and MgCO_3_ predicted by the 1‐D model for local noon at the equator, *L*_*s*_ = 85°. The symbols show Mg^+^ measurements by the Imaging Ultraviolet Spectrograph instrument on Mars Atmosphere and Volatile EvolutioN during two successive orbits.

The major objective of the modeling was to explain the observed Mg^+^ profile and the lack of a detectable Mg signal above the IUVS threshold of 130 cm^−3^ at 90 km (Crismani et al., 2017). These constraints can be met by making the following assumptions in the model (see the discussion in Sections 2.1 and 2.2): When the Mg atoms ablate, hyperthermal collisions with CO_2_ produce Mg^+^ (50%) and MgO or Mg (50%), and the branching ratio for the DR reaction of MgO^+^.(CO_2_)_*n*_ with electrons is set to *β* = 1; that is, the product is MgCO_3_. This is referred to as the “base case.” Figure 7 illustrates a 1‐D model simulation of the major magnesium species—Mg^+^, Mg, and MgCO_3_. The modeled Mg^+^ profile agrees very well—in terms of the top and bottom scale heights and the peak altitude—with that retrieved from the IUV instrument on two successive MAVEN orbits (Crismani et al., 2017). Furthermore, the Mg layer at 90 km is 125 cm^−3^, that is, below the IUVS detection threshold. Note that the concentrations of the other magnesium species treated explicitly in the model—MgO, MgO^+^, Mg^+^.CO_2_, and MgO^+^.CO_2_—are below 1 cm^−3^ between 30 and 200 km.

**Figure 8:**
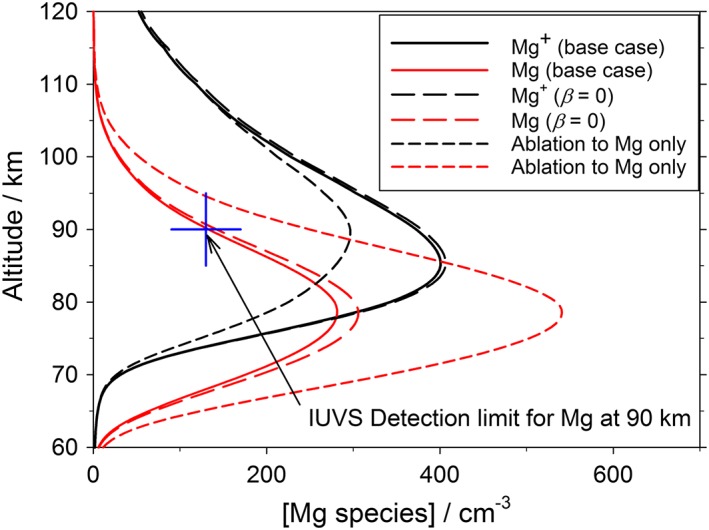
Sensitivity of the 1‐D modeled Mg and Mg^+^ to the branching ratio *β* in reaction 20 and to the fraction of meteoric magnesium which ablates to neutral Mg atoms (see text for further details). The model base case is that illustrated in Figure 6. The blue cross marks the detection limit for Mg atoms at 90 km using Mars Atmosphere and Volatile EvolutioN's Imaging Ultraviolet Spectrograph instrument.

We now consider the sensitivity of the model to the two assumptions described above. Figure 8 illustrates the modeled Mg^+^ and Mg layers when two changes are made to the base case (which is depicted with solid lines in Figure 8). First, when *β* is set to 0 so that MgO^+^.(CO_2_)_*n*_ dissociates to MgO rather than MgCO_3_, there is a modest increase in Mg to 142 cm^−3^ at 90 km, that is, just above the detection limit, and a very small increase of Mg^+^ (because the MgO immediately forms Mg by reaction with O and CO, followed by charge transfer with O_2_^+^ to yield Mg^+^, rather than being permanently removed via MgCO_3_ dimerization). These model runs are shown as long dashed lines in Figure 8. Thus, IUVS observations do not really constrain the value of *β*.

The second change is to assume that all the ablated Mg forms neutral Mg atoms or MgO and no Mg^+^. This model run is depicted with short dashed lines in Figure 8. The model shows almost no sensitivity to whether MgO or Mg is the neutral ablation product, because MgO is reduced rapidly to Mg by reaction with O and CO. Injecting 100% of the magnesium as neutral Mg/MgO causes a very substantial increase in the Mg at 90 km to 254 cm^−3^, that is, almost twice the detection threshold (indicated with a blue cross in Figure 8). Moreover, the Mg^+^ peak falls to around 300 cm^−3^, which is at the low end of the IUVS observations (Figure 7): Increasing the injection rate of magnesium to bring the Mg^+^ peak closer to the observed peak (350–420 cm^−3^) would make the Mg at 90 km even larger. If 70% of the magnesium is injected as neutral species, the Mg at 90 km is 179 cm^−3^; even 60% neutral yields 156 cm^−3^, which should be readily detectable. Thus, it appears that at least 50% of the magnesium needs to be injected as Mg^+^. This implies an unexpectedly high probability of ionization for hyperthermal collisions of Mg with CO_2_, compared to what is expected for Mg colliding with O_2_ and N_2_ (Janches et al., 2017). In the case of Fe, 50% ionization occurs for collisions with CO_2_ at speeds in excess of ~35 km s^−1^ (Thomas et al., 2016), which is relatively high for cosmic dust particles entering the Martian atmosphere. The impact ionization of Mg with CO_2_ should be studied experimentally in the future.

A final point is the sensitivity of the model to the Mg meteoric input function. As discussed in section 2, the absolute injection rate is correlated to the eddy diffusion coefficient, *K*_zz_: A higher injection rate requires faster downward mixing through the mesosphere, in order to reproduce the observed Mg^+^ profile, and vice versa. This means that, although the meteoric input function is uncertain (section 2.1), the amount of available meteoric material in the upper mesosphere above 60 km is quite tightly constrained by the model having to replicate the IUVS measurements of Mg^+^. Hence, the number of metal carbonate cores for nucleating H_2_O‐ice particles (see section 3.2) is also constrained. The lifetime of chemically labile Mg species can be estimated by dividing the sum of the column abundances of Mg^+^, Mg, and MgCO_3_ in Figure 7 by the Mg ablation flux of 1.2 × 10^4^ cm^−2^ s^−1^ (integrated curve in Figure 2). The resulting lifetime of 3.1 days means that the diurnal variation of the meteoric input (which should peak around sunrise) is largely smoothed out (Plane, 2004). Furthermore, the seasonal variability of the meteoric input should be smallest at low latitudes (by analogy with the terrestrial input function; Feng et al., 2013), which is therefore appropriate for modeling the low‐latitude IUVS measurements shown in Figure 7.

### 3.2 Formation of Metal Carbonate H_2_O‐Ice Particles and CO_2_‐Ice Cloud Formation

**Figure 9:**
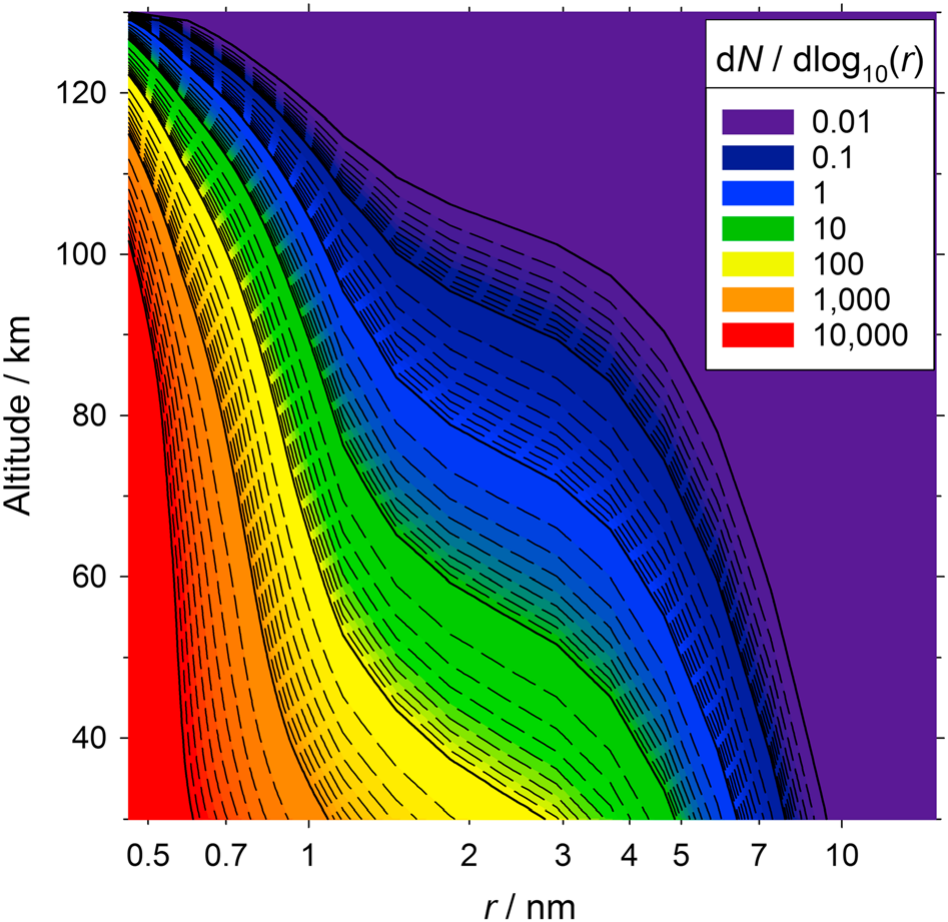
Size distribution of metal‐containing ice particles as a function of height, produced by a meteoric input of 2.7 t sol^−1^.

Figure 9 illustrates the modeled size distribution of H_2_O‐ice particles containing metal carbonate cores, as a function of altitude. In the following discussion these particles are termed nucleating particles (NPs). The fresh supply of new NPs such as MgCO_3_.(H_2_O)_6_ means that the maximum in the distribution at all heights corresponds to monomers (*r* = 0.46 nm), but coagulation leads to an increasing tail to larger NP sizes at lower altitudes. In order to evaluate the formation of mesospheric CO_2_ clouds on the NPs, classical nucleation theory was employed. Nucleation is specifically described here in terms of classical heterogeneous nucleation theory induced by surface diffusion, which has been used previously for the study of mesospheric CO_2_ ice clouds (Listowski et al., 2014; Määttänen et al., 2005, 2007; Nachbar et al., 2016). CO_2_ molecules are assumed to stick and subsequently diffuse to form clusters on the surface of the NPs, which can result in the formation of a critical cluster leading to stable nucleation of CO_2_ ice on the particle. The larger the surface area available for nucleation the greater the likelihood that stable nucleation will occur. The rate of nucleation (*J*_het_, in units of s^−1^) for a spherical NP of radius *r* is given by

$$J_{\mathrm{het}}=A_{N}Z_{\mathrm{het}}\beta_{\mathrm{het}}c_{1,s}\exp\left(\frac{-\Delta F_{\mathrm{het}}}{\mathrm{kT}}\right)$$

where *A*_*N*_ (=4π*r* ^2^) is the surface area of the NP; *Z*_het_ is the heterogeneous Z'eldovich factor (≤1) which allows for super‐critical clusters to dissociate, thereby reducing *J*_het_; *β*_het_ is the flux of CO_2_ molecules from the gas phase to the NP surface; *c*_1,*s*_ is the concentration of monomers on the NP surface from which critical clusters can form; and Δ*F*_het_ is the free energy change involved in forming a critical cluster on the NP surface. As described in Appendix A of Nachbar et al. (2016), Δ*F*_het_ is calculated from the product of the homogeneous free energy of formation of a spherical cluster (calculated from the Gibbs‐Thomson equation) with a factor *f*(*m*,*x*) (Fletcher, 1958), where *x* is the ratio of the size of the NP to the critical cluster and *m* is the contact parameter. For CO_2_ nucleation on these NPs, an *m* value of 0.95 determined by Glandorf et al. (2002) for CO_2_ nucleation on a H_2_O substrate was used. The temperature‐dependent CO_2_ ice density was taken from Mangan et al. (2017), while all other parameters used in this nucleation theory calculation are from Nachbar et al. (2016).

**Figure 10:**
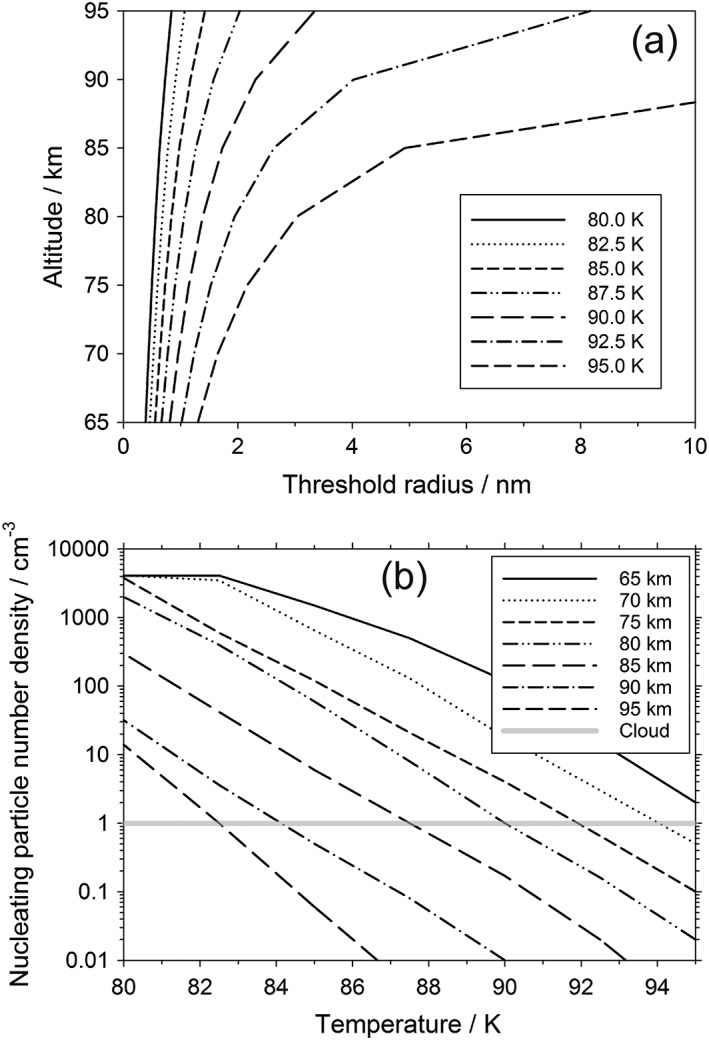
(a) Threshold radius of a H_2_O‐ice nucleating particles (NPs) that can be activated for nucleation and growth of CO_2_‐ice, as a function of height in the Mars atmosphere, and for a selection of temperatures between 80 and 95 K. (b) Number density of NPs with a size equal to or greater than the threshold radius, as a function of temperature, and for a selection of altitudes between 65 and 95 km. The horizontal gray line indicates the number density required to form a CO_2_‐ice cloud with the maximum optical thickness observed.

To assess whether the calculated size distribution of NPs in Figure 9 is sufficient to form CO_2_ clouds, the threshold particle radius necessary for nucleation was evaluated under relevant conditions of altitude (65–100 km) and temperature (80–100 K) using the CO_2_ density profile for the Martian atmosphere (Figure S1). The threshold radius of NPs that produce a nucleation rate of 0.05 s^−1^ was calculated across this range of altitude and temperature, as shown in Figure 10a (see also Table S11). This nucleation rate was chosen to achieve a nucleation probability of 1 on a timescale no longer than 100 s. Any NPs whose size is equal to or larger than this threshold particle radius are assumed to nucleate and produce CO_2_‐ice particles on the several hour timescale of mesospheric cold pockets (Listowski et al., 2014). Note that due to the highly temperature‐dependent nature of the nucleation rate, the timescale over which nucleation occurs is relatively insensitive to NP radius; an order of magnitude increase in nucleation rate only increases the size of the threshold particle radius by 1%.

The concentration of NPs from the size distribution in Figure 9 that are at least as large as the threshold radius is plotted as a function of temperature in Figure 10b, for a range of altitudes between 65 and 95 km. Listowski et al. (2014), using a 1‐D microphysical model for daytime CO_2_ clouds, found that ice particle concentrations of around 1 cm^−3^ were sufficient to reproduce the observed cloud opacities (Määttänen et al., 2010; Vincendon et al., 2011). The horizontal gray line in Figure 10b shows that at 65 km, there are sufficient NPs of threshold radius or larger to produce a CO_2_‐ice cloud with this density of particles at temperatures below 95 K. In contrast, at a height of 95 km the temperature would need to fall below ~82 K to produce such a cloud. At heights between 65 and 80 km where the daytime equatorial clouds tend to be observed (Montmessin et al., 2007), these clouds could potentially form at temperatures below 95 K. The nighttime subtropical clouds that occur above 85 km (Montmessin et al., 2006) would require colder temperatures below 85 K.

A further point to consider is that formation of these metal‐carbonate ice particles only consumes a small amount of the H_2_O available in the mesosphere. For example, at 70 km the total number density of NPs is 4,200 cm^−3^ and the H_2_O concentration locked up in these particles is 32,500 cm^−3^ (an average of 7.7 H_2_O per particle, since most particles are still the MgCO_3_(H_2_O)_6_ monomer). This should be compared with the background H_2_O concentration at 70 km of around 6 × 10^7^ cm^−3^ (http://www-mars.lmd.jussieu.fr/mcd_python/). If all of this H_2_O condensed first onto the NPs before uptake of CO_2_ then, if distributed evenly, there would be a concentration of 4,200 cm^−3^ NPs with a radius of 4.8 nm (assuming an ice density of 950 kg m^−3^). This would greatly facilitate the formation of CO_2_‐ice clouds.

CO_2_‐ice formation on these NPs therefore seems a promising route to cloud formation. However, it needs to be stressed that the calculations presented here assume that the contact parameter, *m*, has a value of 0.95 that is *independent* of temperature below the experimental lower limit of 130 K (Glandorf et al., 2002). If, in fact, *m* decreases with temperature toward the value of *m* = 0.78 measured by Nachbar et al. (2016) on mineral nanoparticles, then the nucleating performance of these NPs would be reduced. For example, at an altitude of 70 km and a temperature of 90 K, if *m* = 0.95, then there would be 18 cm^−3^ NPs of critical radius available for nucleation. If *m* decreases to 0.78 at 90 K, then only 0.7 cm^−3^ NPs would be available, which would be marginal for cloud formation (although this might be offset by the uptake of additional H_2_O, as discussed above). Further experimental measurements of CO_2_ uptake on H_2_O‐ice at temperatures below 130 K would be very desirable.

## 4 Conclusions

In this study we have shown that the IUVS measurements of the persistent Mg^+^ layer around 90 km in the Martian atmosphere can be satisfactorily modeled, assuming a rate of cosmic dust input into the atmosphere of 3 t sol^−1^. The absence of detectable Mg at 90 km suggests that at least 50% of the ablating Mg atoms then ionize through hyperthermal collisions with CO_2_ molecules. This result should be tested experimentally in the future. We have also explored the route of neutralization of Mg^+^ ions via the MgO^+^.(CO_2_)_*n*_ cluster ion, which can undergo DR with electrons to produce MgCO_3_ directly, thus avoiding a buildup of Mg to detectable levels. The IUVS measurements allow the meteoric injection rate of Mg to be constrained, from which the production rate of metal carbonate molecules (principally MgCO_3_ and FeCO_3_) can be determined. These molecules have extremely large electric dipole moments, so that they will immediately form clusters with up to three CO_2_ molecules; these will gradually be replaced with up to six H_2_O molecules at temperatures below 150 K. These clusters should then coagulate efficiently, building up “dirty” ice particles that can act as NPs for the formation of CO_2_‐ice clouds. Formation of observable mesospheric clouds between 65 and 80 km should occur when the temperature drops below 95 K, and clouds above 85 km require temperatures about 5 K colder; these temperatures have been observed by SPICAM on Mars Express (Montmessin et al., 2006). Note, however, that this conclusion depends on the contact angle *m* for CO_2_ nucleation on H_2_O‐ice not varying significantly with temperature below 130 K, although even if *m* does decrease, this may be offset by the initial condensation of additional H_2_O making the NPs larger and hence facilitating CO_2_ uptake.

## Supporting information



Supporting Information S1

Movie S1

Movie S2
