# Supplementary material for: Meteoric Metal Chemistry in the Martian Atmosphere
Source: J Geophys Res Planets. 2018 Mar 6;123(3):695–707. doi: 10.1002/2017JE005510 (PMC5947882; doi:10.1002/2017JE005510)
Supplement: Supplementary file 1 — Supporting Information S1 [file JGRE-123-695-s001.pdf]

AGU PUBLICATIONS

Journal of Geophysical Research - Planets

Supporting Information for

Meteoric Metal Chemistry in the Martian Atmosphere

J. M. C. Plane[1], J. D. Carrillo-Sanchez[1], T. P. Mangan[1], M. Crismani[2], N. M. Schneider[2], and A. Määttänen[3]

[1] School of Chemistry, University of Leeds, Leeds, UK.

[2] Laboratory for Atmospheric and Space Physics (LASP), University of Colorado, USA.

[3] Laboratoire ATmosphères, Milieux, Observations Spatiales (LATMOS), Guyancourt, France.

Contents of this file

Introduction
Figures S1 to S5
Tables S1 to S11

Additional Supporting Information (Files uploaded separately)

Movies S1 to S2

Introduction

Figures S1 to S5, and Tables S1 to S11, are all referred to and their context explained in the main manuscript. Their captions should then be self-explanatory.

Figure S1.

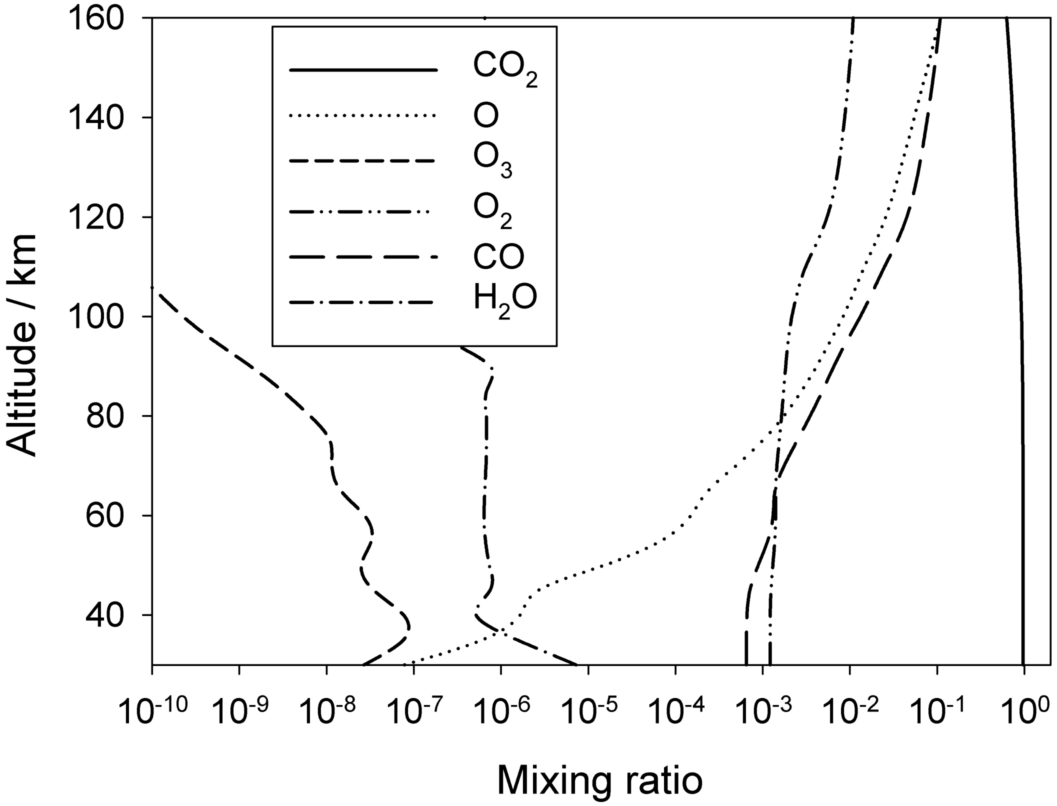

Figure S1. Vertical profiles of atmospheric constituents relevant for the chemistry of Mg^+ and Mg in the atmosphere of Mars. Conditions: local noon at the equator, L_s = 85°. The data is taken from the Mars Climate Database v.5.2 (http://www-mars.lmd.jussieu.fr/mcd_python/) [*Forget et al.*, 1999].

Figure S2.

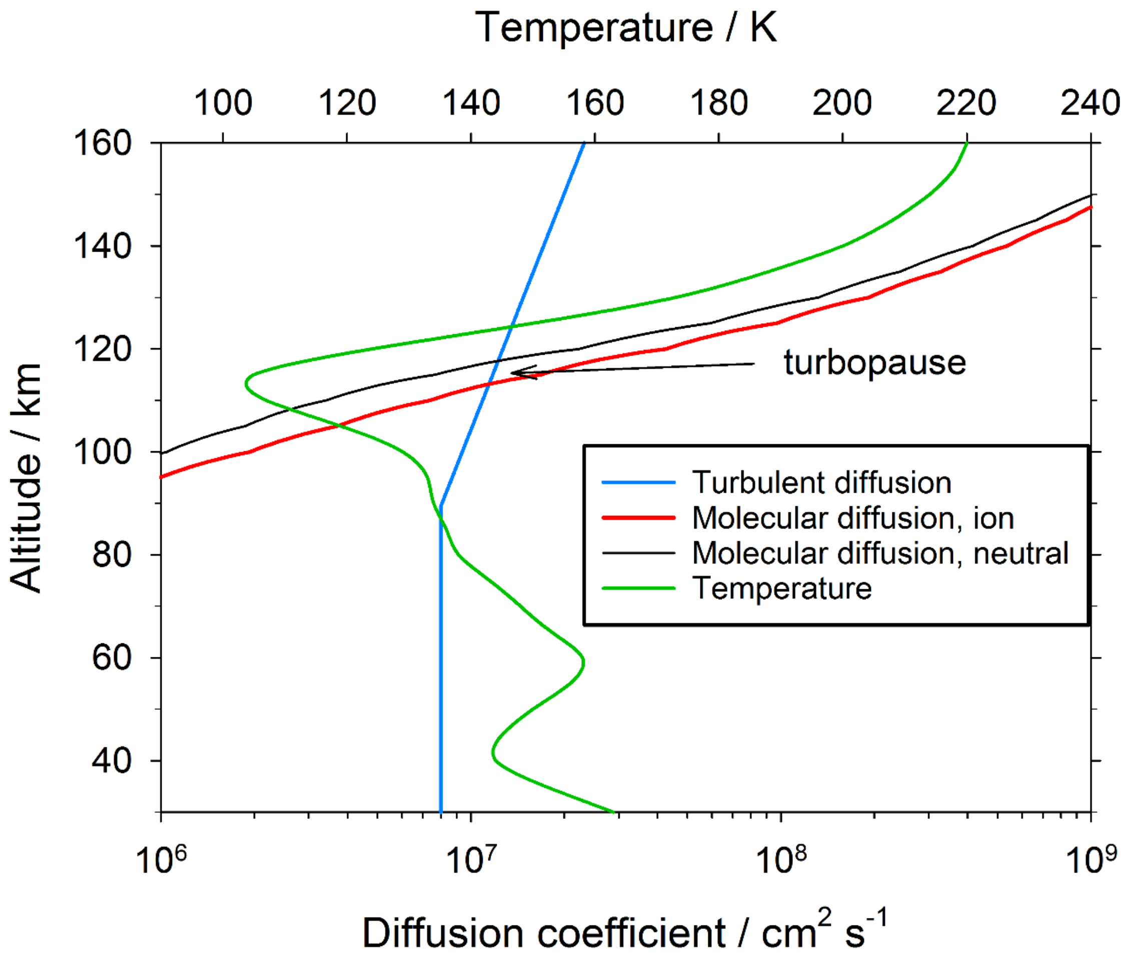

Figure S2. Vertical profiles of the turbulent diffusion coefficient (K_z), ambipolar ion diffusion coefficient, and neutral molecular diffusion coefficient used in the 1D model (see Section 2 in the main paper).

Figure S3.

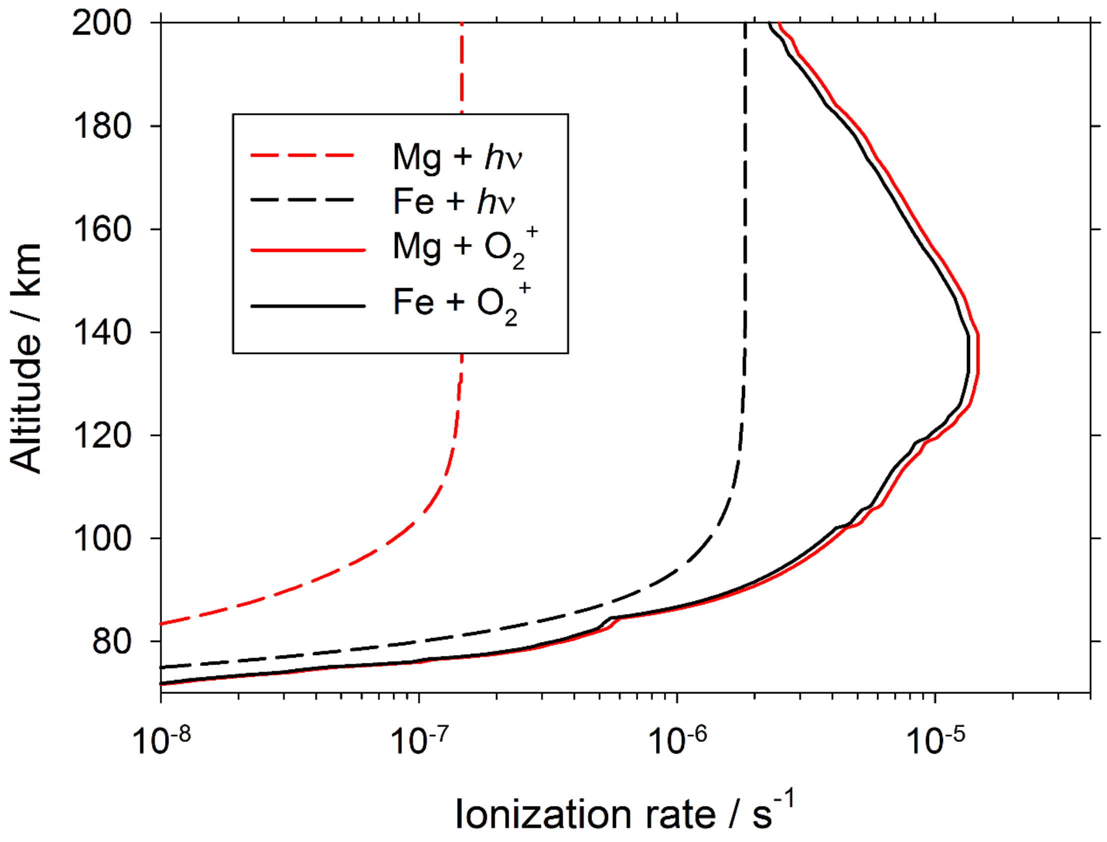

Figure S3. Vertical profiles of the photo-ionization rates of Mg and Fe, and the first-order rates for ionization via charge transfer with the dominant O_2^+ ion. Conditions: local noon, equator, L_s = 85°. The photo-ionization profiles are taken from [*Whalley and Plane*, 2010].

Figure S4.

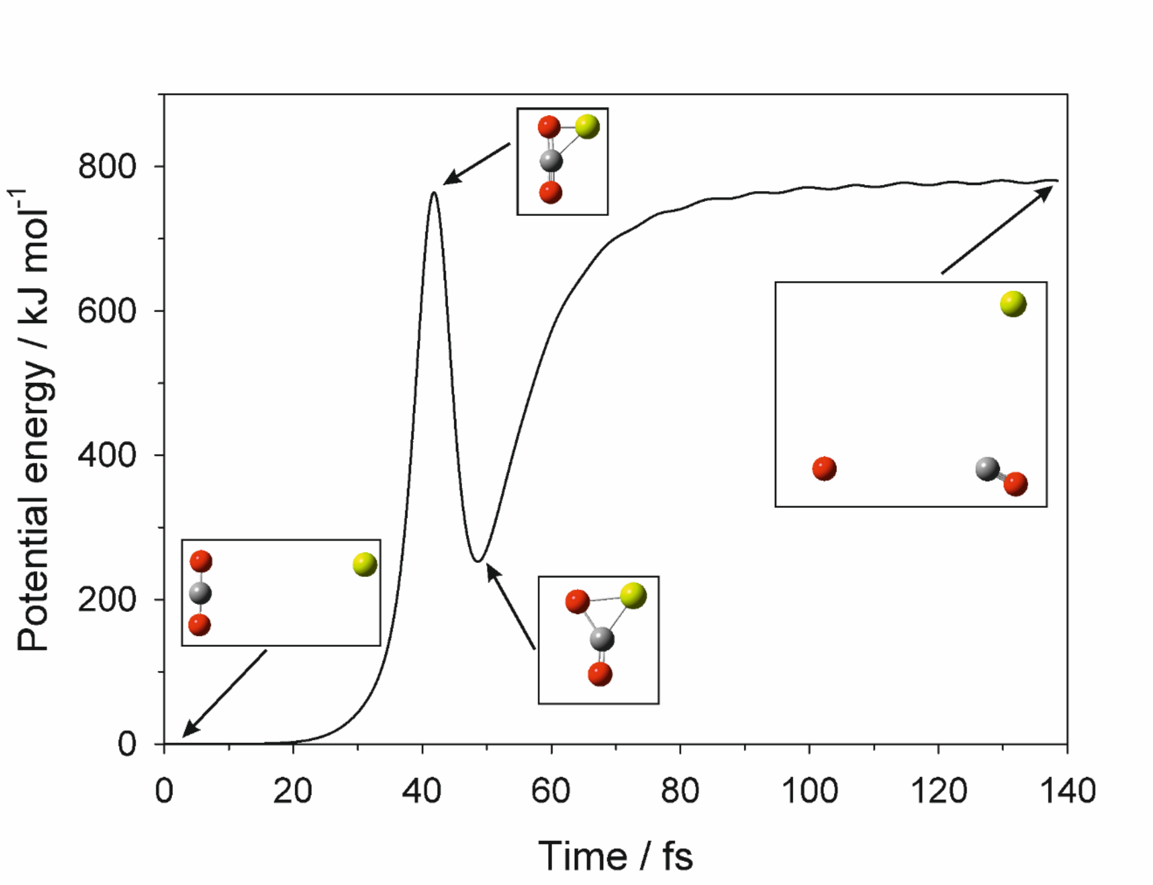

Figure S4. Trajectory calculation of Mg colliding with CO_2 at a relative velocity of 11.9 km s^{-1}, and producing excited Mg(^{3}P), O(^{3}P) and CO. The change in potential energy as a function of time is shown, along with the molecular geometries at four points along the trajectory (Mg: yellow; Oxygen: red; Carbon: grey). Level of theory: B3LYP/6-311+g(2d,p). An animation of the trajectory is included in the SI (movie S1).

Figure S5.

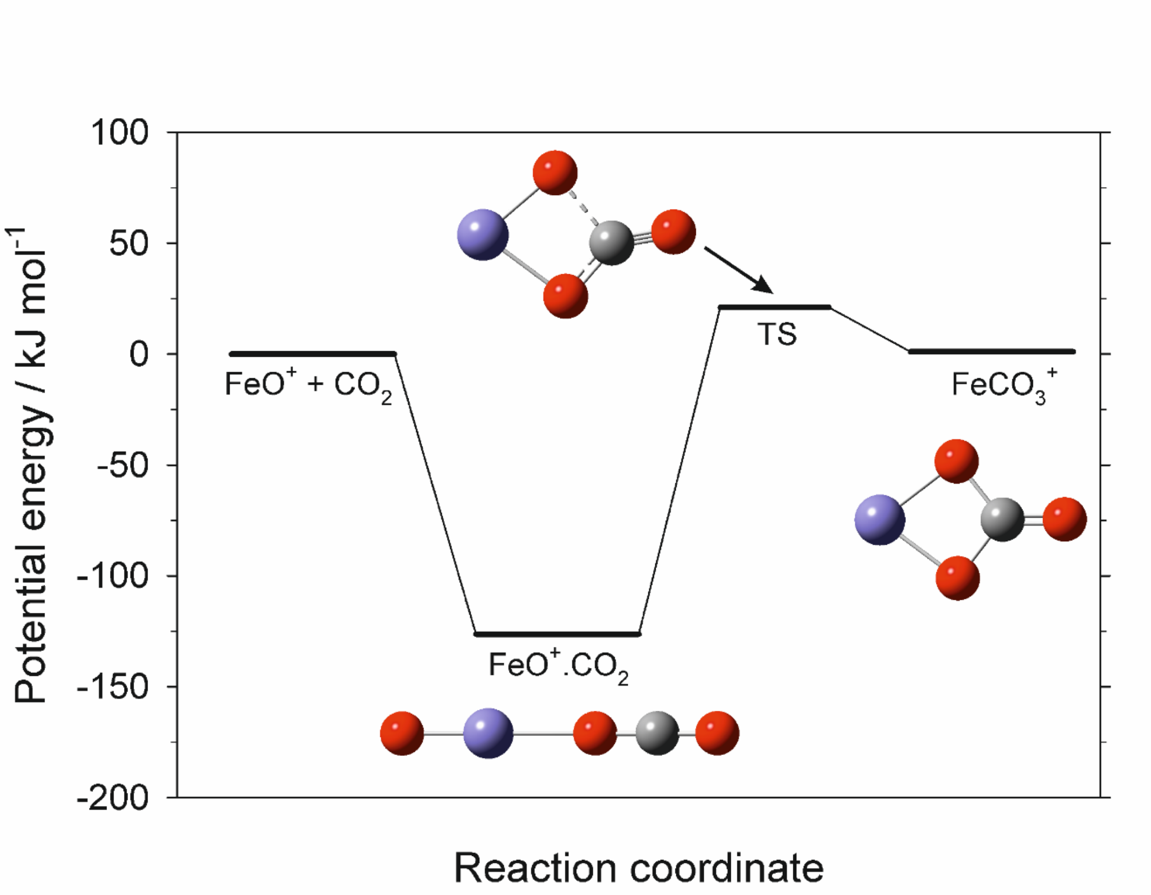

Figure S5. Potential energy surface for the addition of CO_2 to the FeO^+ ion, calculated at the CBS-QB3 level of theory (Fe: blue; O: red; C: grey).

Table S1. Molecular properties of the stationary points on the potential energy surface for $MgO^+(^2\Pi)$ + CO_2 (Figure 4 in the main paper), calculated at the B3LYP/6-311+g(2d,p) level of theory.

Molecule	Geometry (Cartesian co-ordinates in Å)	Rotational constants (GHz)	Vibrational frequencies (cm^{-1})
MgO^+ ($^2\Pi$)	Mg, 0., 0., -0.067<br>O, 0., 0., 1.767	15.655	707
CO_2	C, 0., 0., 0.<br>O, 0., 0., 1.160<br>O, 0., 0., -1.160	11.733	676 (x2), 1364, 2400
$MgO^+.CO_2$ ($^2\Pi$)	Mg, 0., 0., 0.957<br>O, 0., 0., 2.791<br>C, 0., 0., -2.214<br>O, 0., 0., -1.029<br>O, 0., 0., -3.349	1.2900	41 (x2), 109 (x2), 294, 633 (x2), 749, 1382, 2457
TS from $MgO^+.CO_2$ to $MgCO_3^+$	Mg, 1.918, -0.386, -0.124<br>O, 0.526, 0.838, 0.0566<br>C, -0.698, -0.456, 0.027<br>O, -1.808, -0.167, -0.034<br>O, 0.199, -1.327, 0.107	13.011<br>3.4353<br>2.7327	431*i*, 58, 362, 383, 640, 668, 702, 1163, 2171
$MgCO_3^+$ ($^2A_1$)	Mg, 0., -1.715, 0.<br>O, -1.111, -0.119, 0.<br>C, 0., 0.519, 0.<br>O, 0., 1.803, 0.<br>O, 1.111, -0.119, 0.	12.795<br>4.040<br>3.070	163, 354, 495, 510,<br>759, 800, 1046, 1426, 1463

Table S2. Molecular properties of the stationary points on the potential energy surface for FeO^+ ($^6\Sigma$) + CO_2 in Figure S5, calculated at the B3LYP/6-311+g(2d,p) level of theory

Molecule	Geometry (Cartesian co-ordinates in Å)	Rotational constants (GHz)	Vibrational frequencies (cm^{-1})
FeO^+ ($^6\Sigma$)	Mg, 0., 0., -0.067<br>O, 0., 0., 1.767	15.655	707
CO_2	C, 0., 0., 0.<br>O, 0., 0., 1.160<br>O, 0., 0., -1.160	11.733	676 (x2), 1364, 2400
$FeO^+.CO_2$ ($^6\Sigma$)	Fe, 1.022, 0., 0.<br>O, 2.661, 0., 0.<br>C, -2.192, 0., 0.<br>O, -1.000, 0., 0.<br>O, -3.329, 0., 0.	1.1955	42 (x2), 102 (x2),<br>265, 628 (x2), 854, 1382, 2449
TS from $FeO^+.CO_2$ to $FeCO_3^+$	Fe, 1.530, -0.061, 0.<br>O, 0.252, 1.136, 0.031<br>C, -0.847, -0.104, -0.<br>O, -1.974, 0.149, -0.<br>O, -0.043, -1.120, -0.030	12.220<br>2.7500<br>2.2449	445*i*, 145, 380, 406, 690, 700, 767, 1056, 2036
$FeCO_3^+$ ($^6A_1$)	Fe, 0.021, 0., 1.169<br>O, 0., 1.089, -0.294<br>C, -0.028, 0., -1.151<br>O, -0.054, 0., -2.322<br>O, 0., -1.099, -0.290	13.203<br>2.790<br>2.303	143, 392, 435, 567, 746, 751, 795, 863, 1807

Table S3. Molecular properties of $MgCO_3$-$(CO_2)_n$ clusters, calculated at the B3LYP/6-311+g(2d,p) level of theory

Molecule	Geometry (Cartesian co-ordinates in Å)	Rotational constants (GHz)	Vibrational frequencies (cm^{-1})
$MgCO_3$	Mg 0.0000 0.0000 1.5312<br>O 0.0000 1.1375 0.0674<br>C 0.0000 0.0000 -0.7022<br>O 0.0000 0.0000 -1.9050<br>O 0.0000 -1.1375 0.0674	12.2104<br>4.1995<br>3.1248	167 490 530<br>673 806 826<br>903 990 1768
$MgCO_3$-CO_2	Mg -0.0566 -0.0028 1.8070<br>O -0.0163 1.1365 0.3347<br>C 0.0039 0.0073 -0.4386<br>O 0.0366 0.0127 -1.6442<br>O -0.0173 -1.1288 0.3245<br>C -0.0735 0.0478 5.0562<br>O -0.0603 0.0872 6.1989<br>O -0.0887 0.0060 3.8841	12.285<br>0.6134<br>0.5841	35 45 80<br>90 178 193<br>497 553 642<br>645 677 818<br>828 918 1061<br>1388 1759 2446
$MgCO_3$ – $(CO_2)_2$	Mg 1.0230 -1.0223 0.0302<br>O -0.4169 -0.0749 -0.7289<br>C -0.0005 1.0165 -0.0219<br>O -0.5418 2.0948 -0.0495<br>O 1.1086 0.7281 0.7205<br>C 3.7579 -0.2182 -0.0222<br>O 4.4486 0.6782 0.1330<br>O 3.1134 -1.1934 -0.2016<br>C -1.2563 -2.7331 0.1050<br>O -2.3843 -2.7584 -0.0725<br>O -0.0932 -2.7907 0.3109	1.4353<br>0.6302<br>0.4531	21 24 29<br>53 84 93<br>115 154 207<br>260 313 497<br>520 626 642<br>657 657 681<br>807 826 933<br>1091 1343 1345<br>1756 2406 2415
$MgCO_3$ – $(CO_2)_3$	Mg 1.1203 0.9989 1.0762<br>O 2.2720 -0.2336 0.1672<br>C 1.4559 -1.2418 0.5992<br>O 1.6295 -2.4117 0.3511<br>O 0.4325 -0.7483 1.3391<br>C -1.2931 1.1063 2.6978<br>O -2.1128 0.4636 3.1708<br>O -0.4813 1.8275 2.2391<br>C 3.8078 1.1611 2.0385<br>O 4.8238 0.6374 1.9948<br>O 2.8029 1.7726 2.1506<br>C 1.2936 1.5787 -1.7179<br>O 0.8647 2.0952 -0.7455<br>O 1.6728 1.1604 -2.7127	0.6208<br>0.4678<br>0.4119	27 29 34<br>40 44 62<br>74 91 95<br>116 117 191<br>217 237 256<br>305 478 504<br>619 622 653<br>657 661 661<br>680 789 832<br>946 1114 1341<br>1343 1353 1749<br>2395 2402 2420
$MgCO_3$ – $(CO_2)_4$	Mg 0.0104 -0.0047 0.4603<br>O 0.3911 -1.0285 -1.1193<br>C -0.0450 0.0147 -1.8731	0.4380<br>0.3703<br>0.3128	20 23 28<br>31 33 44<br>59 61 74

	O	-0.0730	0.0251	-3.0832		80	96	100
	O	-0.4454	1.0448	-1.0823		112	121	130
	C	-0.0697	2.8415	1.3080		179	194	202
	O	-0.1222	3.9222	0.9305		230	235	253
	O	-0.0126	1.7542	1.7508		461	495	616
	C	2.8811	-0.1904	-0.2548		627	648	657
	O	2.2519	0.1654	0.6752		658	660	664
	O	3.5752	-0.5097	-1.1096		667	678	780
	C	0.1299	-2.8636	1.2664		838	953	1125
	O	0.0948	-1.7815	1.7238		1347	1347	1359
	O	0.1637	-3.9397	0.8739		1360	1738	2390
	C	-2.8903	0.1893	-0.1139		2395	2410	2428
	O	-3.6245	0.5212	-0.9296				
	O	-2.2177	-0.1805	0.7794				

Table S4. Molecular properties of $FeCO_3$-$(CO_2)_n$ clusters, calculated at the B3LYP/6-311+g(2d,p) level of theory

Molecule	Geometry (Cartesian co-ordinates in Å)				Rotational constants (GHz)	Vibrational frequencies (cm^{-1})		
$FeCO_3$	Fe	0.0000	0.0000	1.5813	12.8179	158	384	466
	O	0.0000	1.1102	0.0691	2.7544	636	792	827
	C	0.0000	0.0000	-0.7322	2.2672	915	1022	1800
	O	0.0000	0.0000	-1.9284				
	O	0.0000	-1.1102	0.0691				
$FeCO_3$-CO_2	x							
$FeCO_3$ – $(CO_2)_2$	Fe	-0.0005	-0.2393	-0.0066	1.1872	18	21	22
	O	-1.1039	1.3120	0.0259	0.5976	23	65	79
	C	0.0021	2.1020	0.0007	0.3975	93	124	161
	O	0.0033	3.3023	0.0043		168	206	372
	O	1.1063	1.3098	-0.0292		425	642	655
	C	2.8829	-1.2061	0.0079		657	658	658
	O	3.9772	-0.8643	0.0118		779	810	942
	O	1.7735	-1.5962	0.0041		1075	1352	1354
	C	-2.8850	-1.2034	-0.0005		1790	2400	2411
	O	-3.9791	-0.8611	0.0065				
	O	-1.7758	-1.5940	-0.0081				
$FeCO_3$ – $(CO_2)_3$	Fe	1.4273	0.5168	0.9756	0.4884	10	20	22
	O	1.5041	-0.8246	-0.3944	0.4602	42	45	54
	C	0.7661	-1.6671	0.3695	0.3246	65	69	76
	O	0.4275	-2.7782	0.0594		87	93	110
	O	0.4706	-1.0489	1.5389		136	158	167
	C	-0.9844	1.0305	2.8701		189	360	418
	O	-1.9115	0.5002	3.2931		640	651	651
	O	-0.0556	1.6213	2.4631		654	657	664
	C	3.9787	2.0046	2.4401		665	772	815
	O	4.9996	2.1539	2.9479		953	1094	1354
	O	2.9385	1.8685	1.9255		1354	1372	1781
	C	1.5731	1.5756	-1.9477		2393	2398	2424
	O	1.7103	2.0004	-0.8624				
	O	1.4443	1.2133	-3.0303				
$FeCO_3$ – $(CO_2)_4$	Fe	0.5418	-0.0034	-0.0195	0.4076	15	17	21
	O	1.1188	-1.6814	0.7281	0.3105	31	36	40
	C	-0.0962	-2.2272	0.4997	0.2558	40	51	55
	O	-0.4222	-3.3558	0.7555		57	68	75
	O	-0.8949	-1.2865	-0.0689		84	92	102
	C	-1.2082	0.1371	-2.6332		108	118	142
	O	-1.9993	-0.4698	-3.2057		155	174	192
	O	-0.4023	0.7890	-2.0861		355	417	642
	C	3.5835	-0.6287	-0.2572		646	652	654
	O	2.8588	0.2802	-0.4164		654	658	664

	O	4.3357	-1.4864	-0.1212			665	674	771
	C	0.0375	3.0545	0.9011			818	956	1101
	O	0.6368	2.1616	0.4406			1355	1357	1363
	O	-0.5228	3.9519	1.3494			1370	1780	2392
	C	-2.9452	0.2444	1.2778			2395	2405	2423
	O	-3.6575	-0.6157	1.5659					
	O	-2.2600	1.1505	1.0077					

Table S5. Molecular properties of $MgCO_3$-$(H_2O)_n$ clusters, calculated at the B3LYP/6-311+g(2d,p) level of theory

Molecule	Geometry (Cartesian co-ordinates in Å)				Rotational constants (GHz)	Vibrational frequencies (cm^{-1})		
$MgCO_3$								
$MgCO_3$-H_2O	Mg	-0.0050	0.2016	0.0000	11.6731	11	17	81
	O	-1.8318	-0.1613	0.0000	0.6203	94	178	196
	C	-2.0203	1.1940	0.0000	0.5890	497	553	644
	O	-3.1016	1.7281	0.0000		645	677	818
	O	-0.8298	1.8705	0.0000		828	918	1061
	C	2.6397	-1.6630	0.0000		1386	1759	2445
	O	3.4855	-2.4325	0.0000				
	O	1.7783	-0.8664	0.0000				
$MgCO_3$ – $(H_2O)_2$	Mg	2.3938	0.8532	-0.1573	2.7853	55	61	94
	O	1.1728	-0.5197	-0.6963	1.8274	125	164	273
	C	0.2294	0.0544	0.1008	1.2079	281	322	378
	O	-0.8970	-0.3612	0.2350		493	513	548
	O	0.7228	1.1589	0.7268		561	674	718
	O	3.7173	-0.6842	-0.0052		754	781	833
	H	2.9434	-1.2790	-0.1648		946	1110	1592
	H	4.3064	-1.0865	0.6423		1594	1753	3430
	O	2.3510	2.8640	-0.4641		3438	3863	3864
	H	2.3903	3.4966	-1.1897				
	H	1.4311	2.8314	-0.1016				
$MgCO_3$ – $(H_2O)_3$	Mg	0.9613	0.1481	-0.5081	1.9398	55	73	85
	O	-0.6143	-1.0159	-0.9610	1.2652	110	122	152
	C	-1.4047	-0.1387	-0.2601	1.1159	208	254	271
	O	-2.6006	-0.2668	-0.1343		284	314	359
	O	-0.6657	0.8540	0.2572		389	453	466
	O	1.5656	-1.6714	0.2756		496	562	575
	H	0.6360	-1.9289	0.0168		647	678	743
	H	1.7148	-1.9356	1.1901		787	830	865
	O	1.7899	1.8801	0.2681		965	1152	1598
	H	2.2776	2.6315	-0.0858		1611	1616	1748
	H	0.9064	2.1898	0.5542		3243	3306	3612
	O	1.1465	0.0098	-2.5649		3861	3872	3873
	H	1.7308	-0.4219	-3.1965				
	H	0.3042	-0.5159	-2.4875				
$MgCO_3$ – $(H_2O)_4$	Mg	1.1250	0.4133	0.2885	1.2416	39	71	76
	O	-0.3971	-0.7711	0.0835	1.0965	82	120	139
	C	-1.2160	-0.0009	0.8312	0.7643	159	194	215
	O	-2.3820	-0.2371	1.0479		229	242	280
	O	-0.5387	1.0767	1.2961		309	339	352
	O	0.5925	-2.3431	-1.6784		359	393	447
	H	-0.0671	-1.9145	-1.0576		473	515	552

	H	0.6022	-3.2824	-1.4670		570	672	678
	O	0.8292	2.3363	-0.4775		742	765	828
	H	0.5814	2.6183	-1.3645		860	936	989
	H	0.0038	2.2853	0.0817		1068	1165	1582
	O	2.3682	-0.6583	-0.8880		1612	1643	1695
	H	1.8032	-1.4138	-1.2770		1753	2796	3161
	H	3.0950	-0.4628	-1.4852		3249	3414	3866
	O	1.8603	0.6727	2.2058		3869	3885	3898
	H	2.5018	1.2008	2.6904				
	H	0.9427	0.9421	2.4595				
$MgCO_3$ – $(H_2O)_5$	Mg	-0.0104	-0.7563	0.0021	0.8966	49	53	58
	O	0.5978	1.0621	0.6093	0.7793	71	90	96
	C	-0.4601	1.6260	-0.0839	0.7550	124	158	171
	O	-0.6635	2.8200	-0.1022		217	261	271
	O	-1.1636	0.6838	-0.6846		284	290	313
	O	2.9418	0.8878	-0.5120		327	331	338
	H	2.1149	1.2115	-0.0422		359	375	413
	H	3.1383	1.5261	-1.2057		428	480	507
	O	-0.2570	-1.5987	1.8391		537	542	646
	H	-0.1183	-0.8579	2.5169		670	686	730
	H	-0.9936	-2.1465	2.1268		746	831	853
	O	1.7581	-1.3769	-0.8178		907	950	1041
	H	2.3520	-0.5509	-0.8148		1118	1201	1628
	H	2.2936	-2.1187	-0.5187		1655	1664	1698
	O	-1.4127	-1.9042	-1.1156		1710	1754	2836
	H	-1.2580	-2.4370	-1.9033		2909	3095	3153
	H	-1.8556	-1.0802	-1.4001		3630	3867	3869
	O	0.3263	0.5471	3.1546		3870	3874	3883
	H	0.4730	0.9901	2.2660				
	H	-0.2654	1.1194	3.6541				
$MgCO_3$ – $(H_2O)_6$	Mg	-0.8409	0.1584	-0.0734	0.7724	33	48	52
	O	1.1873	0.0554	0.1579	0.5966	65	70	85
	C	1.3501	0.3040	-1.1762	0.5455	103	115	154
	O	2.4293	0.3891	-1.7201		177	203	221
	O	0.1601	0.4383	-1.7512		261	269	276
	O	1.2489	-2.3677	1.1363		292	306	319
	H	1.5008	-1.4801	0.7489		320	324	333
	H	1.6943	-3.0394	0.6092		348	364	368
	O	-1.1062	1.6646	1.2825		430	439	481
	H	-0.2047	2.0022	1.5824		489	520	527
	H	-1.7104	2.4084	1.2024		633	669	675
	O	-1.2175	-1.6273	0.8828		716	756	815
	H	-0.3208	-2.0782	1.0177		844	869	877
	H	-1.7096	-1.7220	1.7047		985	1011	1069
	O	-2.6668	0.3437	-1.0484		1099	1209	1644
	H	-3.3645	-0.3180	-1.0020		1654	1660	1698

	H	-2.4605	0.4857	-2.0258		1699	1704	1755
	O	1.3975	2.0489	1.8527		2915	2980	3012
	H	1.5850	1.3009	1.2160		3165	3211	3266
	H	1.9717	2.7781	1.5964		3867	3870	3872
	O	-1.7418	0.5833	-3.4827		3873	3876	3887
	H	-0.8209	0.5522	-3.1037				
	H	-1.7903	1.3678	-4.0381				
$MgCO_3$ – $(H_2O)_7$	Mg	-0.3475	-0.7353	-0.2305	0.5995	24	43	47
	O	1.1814	0.6914	-0.2466	0.5433	59	71	79
	C	0.2148	1.5971	-0.4858	0.4773	97	104	129
	O	0.3142	2.7864	-0.1845		150	170	174
	O	-0.8611	1.0295	-0.9874		201	204	215
	O	2.4012	-0.5814	-2.2405		235	247	280
	H	2.1104	0.0817	-1.5572		286	293	305
	H	2.4459	-0.1148	-3.0819		325	337	339
	O	0.1505	-1.2472	1.6904		364	372	389
	H	0.8196	-0.6117	2.0954		416	450	458
	H	-0.5234	-1.4507	2.3468		483	504	510
	O	0.4500	-2.1095	-1.5029		514	622	657
	H	1.2359	-1.6423	-1.9357		670	694	718
	H	0.7371	-2.9952	-1.2582		744	769	812
	O	-2.3196	-1.2422	0.1742		842	870	881
	H	-2.8701	-1.8733	-0.2999		1017	1026	1087
	H	-2.8312	-0.3545	0.1960		1167	1276	1633
	O	1.9595	0.5407	2.2644		1654	1673	1682
	H	1.8555	0.8182	1.3126		1692	1695	1717
	H	1.7936	1.3318	2.7889		1742	2732	2949
	O	-3.3235	1.1244	-0.0006		3009	3235	3279
	H	-2.5530	1.3380	-0.5803		3393	3439	3651
	H	-3.1877	1.7745	0.7151		3862	3867	3872
	O	-1.9493	3.1927	1.3776		3875	3875	3880
	H	-1.1640	3.1751	0.7798				
	H	-2.2004	4.1188	1.4472				
$MgCO_3$ – $(H_2O)_8$	Mg	0.4785	0.6287	0.8644	0.5180	23	41	42
	O	0.2446	0.6884	-1.1779	0.4438	52	58	67
	C	-0.3314	-0.5021	-1.1209	0.3948	79	92	102
	O	-1.1167	-0.9460	-1.9574		105	127	152
	O	0.0045	-1.1493	0.0024		166	179	188
	O	3.1357	-0.0723	-1.1421		205	222	225
	H	2.3433	0.2729	-1.5902		234	261	265
	H	2.9807	-1.0462	-1.1033		285	291	308
	O	-0.3045	2.5265	1.0214		316	333	343
	H	-0.6599	2.8618	0.1431		347	358	387
	H	-0.9443	2.7325	1.7105		420	437	457
	O	2.4659	0.7827	1.1754		458	476	508
	H	2.8859	0.4577	0.2994		515	531	614

	H	2.9447	1.5643	1.4679		632	665	673
	O	-0.4899	0.1357	2.6338		690	713	740
	H	-0.0545	-0.1302	3.4502		755	783	795
	H	-1.1668	-0.5953	2.4042		868	876	945
	O	-0.9698	2.9984	-1.4658		1012	1022	1144
	H	-0.5899	2.0968	-1.6500		1160	1269	1625
	H	-1.8611	2.9989	-1.8303		1653	1668	1678
	O	-1.9529	-1.7885	1.7439		1685	1691	1714
	H	-1.2663	-1.9017	1.0473		1717	1741	2738
	H	-2.7444	-1.6069	1.1982		2778	3057	3274
	O	-3.4875	-1.2753	-0.5554		3403	3421	3464
	H	-2.7019	-1.2496	-1.1502		3488	3609	3690
	H	-4.1193	-1.8639	-0.9796		3869	3870	3872
	O	2.2171	-2.6191	-0.7179		3874	3879	3880
	H	1.3403	-2.2746	-0.4318				
	H	2.0366	-3.2808	-1.3937				
$MgCO_3$ –	Mg	0.6805	0.7040	0.7433	0.5202	32	36	43
$(H_2O)_9$	O	0.7445	0.2841	-1.2810	0.3434	56	60	67
	C	-0.0724	-0.7210	-1.0743	0.3189	70	89	97
	O	-0.7970	-1.2361	-1.9355		103	112	115
	O	-0.0874	-1.1039	0.2099		140	156	162
	O	3.3495	-0.9183	-0.6036		178	188	211
	H	2.6913	-0.5649	-1.2298		227	233	246
	H	2.9935	-1.8046	-0.3614		257	263	270
	O	0.1217	2.6195	0.3848		284	294	307
	H	-0.2127	2.7351	-0.5792		319	324	332
	H	-0.4580	3.1120	0.9740		353	354	397
	O	2.6137	0.6014	1.3194		421	431	462
	H	3.0581	0.0018	0.6179		472	475	478
	H	3.2004	1.3426	1.4991		521	525	559
	O	-0.4465	0.7283	2.5085		605	628	648
	H	-0.1088	0.4321	3.3606		660	668	683
	H	-1.2711	0.1806	2.3161		737	751	768
	O	-0.6101	2.5656	-2.0619		812	817	878
	H	-0.1368	1.7156	-2.1847		887	928	937
	H	-1.5649	2.3476	-2.1477		1024	1081	1107
	O	-2.3123	-0.9111	1.6724		1160	1292	1611
	H	-1.6062	-1.2628	1.0771		1655	1670	1681
	H	-3.0146	-0.6727	1.0480		1685	1688	1704
	O	-3.3024	-0.7812	-1.1803		1718	1720	1735
	H	-2.4021	-1.0843	-1.4910		2686	2752	3012
	H	-3.9289	-1.4439	-1.4898		3162	3392	3434
	O	1.8570	-3.0671	0.2667		3458	3493	3509
	H	1.0386	-2.5225	0.3034		3557	3672	3770
	H	1.6342	-3.8489	-0.2493		3865	3867	3872
	O	-3.2999	1.8490	-2.1000		3873	3878	3879

	H	-3.3886	0.9107	-1.8208				
	H	-3.7477	1.9153	-2.9496				
$MgCO_3$ – $(H_2O)_{10}$	Mg	-0.4035	1.3165	0.4527	0.3940	30	33	50
	O	-0.4808	-0.6436	1.0796	0.3363	55	63	67
	C	-0.2941	-1.0544	-0.1547	0.2847	76	80	86
	O	-0.3084	-2.2592	-0.4893		97	98	120
	O	-0.0909	-0.0713	-1.0060		128	140	150
	O	-3.2200	-0.6983	1.4602		156	163	168
	H	-2.2881	-0.9243	1.6570		172	188	189
	H	-3.4404	-1.3551	0.7654		196	209	230
	O	0.8130	1.8820	1.9771		239	243	249
	H	1.3468	1.0756	2.2902		257	259	278
	H	1.3785	2.6595	2.0044		291	301	324
	O	-2.3880	1.6217	0.7114		330	335	344
	H	-2.8653	0.7618	0.9805		419	424	431
	H	-2.9062	2.3781	0.9985		447	457	470
	O	0.1553	2.8759	-0.7895		487	502	511
	H	-0.3225	2.7176	-1.6367		537	584	609
	H	1.0877	2.6433	-0.9958		627	631	666
	O	1.9066	-0.3929	2.5262		676	690	705
	H	1.0918	-0.8401	2.2207		724	745	763
	H	2.5737	-0.6135	1.8435		825	832	857
	O	2.3617	1.2768	-1.2827		876	898	924
	H	1.6660	0.6092	-1.4335		973	1053	1054
	H	3.0251	0.7990	-0.7581		1108	1328	1575
	O	2.2681	-2.7183	-1.2003		1646	1658	1666
	H	1.2984	-2.6573	-1.0077		1675	1688	1693
	H	2.3442	-2.6320	-2.1564		1707	1728	1731
	O	-3.0767	-2.7175	-0.4848		1738	2810	2871
	H	-2.0961	-2.7218	-0.5404		3232	3357	3454
	H	-3.3307	-3.6214	-0.2731		3489	3511	3552
	O	3.5830	-0.8865	0.2860		3555	3557	3586
	H	3.1447	-1.5899	-0.2648		3604	3625	3727
	H	4.4864	-1.1884	0.4299		3864	3868	3876
	O	-1.0031	1.6305	-2.9338		3883	3884	3898
	H	-1.8860	1.5753	-3.3126				
	H	-0.8338	0.7780	-2.4815				
$MgCO_3$ – $(H_2O)_6$ dimer	Mg	-2.3920	0.1699	-0.4645	0.2450	36	44	50
	O	-1.7754	-1.6428	0.4846	0.1761	55	60	71
	C	-1.0634	-0.9144	1.3149	0.1385	72	78	86
	O	-0.2046	-1.3939	2.0963		91	96	100
	O	-1.2989	0.3681	1.2167		102	111	111
	O	-0.1970	-2.6301	-1.6120		123	139	146
	H	-0.7438	-2.5671	-0.8006		151	159	168
	H	0.3741	-1.8244	-1.5533		171	177	182
	O	-4.3819	-0.1664	-0.2145		187	197	201

	H	-4.5467	-1.0421	0.2429		202	222	234
	H	-5.0698	-0.0200	-0.8711		243	248	255
	O	-1.7680	-0.3807	-2.3300		256	262	263
	H	-1.4137	-1.2904	-2.3744		269	270	274
	H	-0.9735	0.2131	-2.4753		276	288	292
	O	-2.2759	2.1326	-1.0473		304	307	324
	H	-1.4042	2.2080	-1.4931		326	330	330
	H	-2.2956	2.8682	-0.3854		422	423	451
	O	-4.2724	-2.5013	0.9605		452	454	457
	H	-3.2873	-2.4168	0.9476		461	474	484
	H	-4.5242	-2.6903	1.8705		484	576	579
	O	-1.9391	4.1027	0.7752		603	609	635
	H	-2.5349	4.4024	1.4689		636	672	678
	H	-1.1457	3.7257	1.2238		695	697	719
	Mg	2.3697	-0.2027	0.5068		729	757	758
	O	1.7537	1.6096	-0.4426		776	782	801
	C	1.0415	0.8812	-1.2730		803	828	829
	O	0.1828	1.3610	-2.0542		862	867	890
	O	1.2767	-0.4012	-1.1746		900	932	944
	O	4.2509	2.4677	-0.9194		969	972	1002
	H	3.2659	2.3838	-0.9066		1002	1059	1064
	H	4.5030	2.6564	-1.8294		1086	1090	1334
	O	1.7461	0.3473	2.3725		1360	1549	1573
	H	1.3923	1.2571	2.4179		1643	1644	1663
	H	0.9514	-0.2465	2.5174		1663	1684	1684
	O	4.3595	0.1343	0.2582		1709	1710	1714
	H	4.5246	1.0094	-0.2003		1716	1756	1757
	H	5.0482	-0.0124	0.9138		3110	3123	3126
	O	2.2541	-2.1652	1.0892		3127	3314	3316
	H	1.3826	-2.2416	1.5351		3362	3370	3390
	H	2.2750	-2.9012	0.4279		3392	3436	3437
	O	0.1758	2.5976	1.6539		3518	3522	3542
	H	0.7229	2.5339	0.8426		3554	3613	3614
	H	-0.3954	1.7921	1.5954		3871	3871	3879
	O	1.9178	-4.1358	-0.7326		3880	3882	3882
	H	2.5126	-4.4395	-1.4254				
	H	1.1250	-3.7586	-1.1821				

Table S6. Molecular properties of $FeCO_3$-$(H_2O)_n$ clusters, calculated at the B3LYP/6-311+g(2d,p) level of theory

Molecule	Geometry (Cartesian co-ordinates in Å)				Rotational constants (GHz)	Vibrational frequencies (cm^{-1})		
$FeCO_3$	Fe	0.0000	0.0000	1.5813	12.8179	158	384	466
	O	0.0000	1.1102	0.0691	2.7544	636	792	827
	C	0.0000	0.0000	-0.7322	2.2672	915	1022	1800
	O	0.0000	0.0000	-1.9284				
	O	0.0000	-1.1102	0.0691				
$FeCO_3$-H_2O	Fe	-0.0645	0.0005	-0.1461	11.2910	10	18	71
	O	-1.5709	1.1052	0.0203	0.6084	76	164	192
	C	-2.3612	-0.0004	0.1078	0.5848	378	442	636
	O	-3.5519	-0.0009	0.2407		642	646	795
	O	-1.5701	-1.1054	0.0192		803	929	1056
	C	3.1609	-0.0001	0.0493		1377	1798	2433
	O	4.2644	-0.0011	0.3610				
	O	2.0382	0.0009	-0.2841				
$FeCO_3$ – $(H_2O)_2$	Fe	-0.9081	-0.5963	0.1387	4.1868	39	60	79
	O	0.6658	0.2746	0.6926	1.2504	144	182	192
	C	1.4394	-0.4780	-0.1325	1.0069	236	307	332
	O	2.6362	-0.4006	-0.2232		352	395	451
	O	0.6449	-1.3208	-0.8285		500	564	650
	O	-2.9881	-0.3213	-0.0288		686	771	814
	H	-3.4113	0.5132	-0.2659		956	1100	1621
	H	-3.3897	-1.0248	-0.5582		1652	1779	3530
	O	-1.5461	-2.7165	-0.4198		3767	3844	3870
	H	-1.6056	-3.4131	0.2454				
	H	-0.6333	-2.7614	-0.7805				
$FeCO_3$ – $(H_2O)_3$	Fe	0.8198	0.6867	-0.4205	1.4881	25	31	77
	O	-0.4351	-0.7814	-0.0984	1.3775	93	128	160
	C	-1.2449	0.0266	0.6358	0.7199	196	237	246
	O	-2.2756	-0.3134	1.1558		258	301	338
	O	-0.6963	1.2530	0.6764		351	378	437
	O	0.8931	-2.6401	-1.3614		460	482	628
	H	0.1630	-2.2100	-0.8455		663	675	766
	H	1.1687	-3.4145	-0.8594		813	844	979
	O	1.1576	2.8337	-0.3716		1099	1139	1621
	H	1.1742	3.4664	-1.0994		1641	1688	1780
	H	0.3322	2.9805	0.1285		2800	3334	3651
	O	2.2211	-0.4391	-1.4325		3855	3865	3868
	H	1.8299	-1.3810	-1.4653				
	H	2.6399	-0.2490	-2.2784				
$FeCO_3$ – $(H_2O)_4$	Fe	-0.0020	-0.6221	0.0084	1.3476	32	48	68
	O	1.0365	1.0186	-0.3492	0.8844	77	92	110
	C	0.0006	1.8111	0.0011	0.5420	161	205	224

	O	0.0004	3.0145	-0.0024		255	281	306
	O	-1.0364	1.0211	0.3565		331	333	344
	O	3.4207	0.0323	0.0671		357	375	395
	H	2.6956	0.6957	-0.0771		429	479	481
	H	4.0658	0.1674	-0.6349		653	660	665
	O	-3.4160	0.0333	-0.0862		763	810	862
	H	-4.0734	0.1716	0.6037		864	987	1088
	H	-2.6933	0.6974	0.0666		1114	1153	1638
	O	1.6712	-1.8671	0.1291		1638	1688	1689
	H	2.4717	-1.2424	0.0912		1783	2861	2884
	H	1.8198	-2.4979	0.8422		3303	3321	3856
	O	-1.6708	-1.8701	-0.1349		3856	3868	3869
	H	-2.4716	-1.2461	-0.1040				
	H	-1.8103	-2.4980	-0.8523				
$FeCO_3$ –	Fe	0.0210	-0.7851	-0.0768	0.8602	40	47	57
$(H_2O)_5$	O	0.5803	1.1716	0.5693	0.7289	60	79	92
	C	-0.4862	1.6571	-0.1160	0.6993	99	127	140
	O	-0.7915	2.8251	-0.1722		204	222	263
	O	-1.1393	0.6501	-0.7081		266	273	290
	O	2.9902	0.9134	-0.4353		294	321	330
	H	2.1410	1.2634	-0.0403		339	348	375
	H	3.2470	1.5217	-1.1361		385	415	469
	O	-0.1583	-1.6568	1.8307		529	536	627
	H	-0.0505	-0.9051	2.4926		665	671	708
	H	-0.9461	-2.1601	2.0620		740	804	844
	O	1.8995	-1.4025	-0.7695		880	982	1028
	H	2.4671	-0.5641	-0.7266		1113	1169	1642
	H	2.3952	-2.1186	-0.3574		1647	1661	1682
	O	-1.6373	-1.9536	-1.1376		1695	1758	2899
	H	-1.4797	-2.3634	-1.9964		3007	3174	3227
	H	-2.0767	-1.1031	-1.3204		3674	3854	3857
	O	0.3435	0.5465	3.1144		3861	3870	3873
	H	0.4523	1.0135	2.2381				
	H	-0.2336	1.0967	3.6536				
$FeCO_3$ –	Fe	-0.8687	0.1901	-0.0103	0.7333	28	43	50
$(H_2O)_6$	O	1.2228	0.0710	0.2444	0.5681	61	67	78
	C	1.4013	0.2994	-1.0852	0.5394	92	105	125
	O	2.4798	0.3660	-1.6293		183	187	208
	O	0.2117	0.4364	-1.6707		240	263	276
	O	1.2807	-2.4224	1.0823		287	300	304
	H	1.5238	-1.5019	0.7842		307	325	335
	H	1.7941	-3.0317	0.5412		341	364	378
	O	-1.1127	1.8262	1.2625		386	398	468
	H	-0.2007	2.1308	1.5663		493	521	535
	H	-1.6188	2.5924	0.9706		634	661	680
	O	-1.2050	-1.8012	0.6608		715	738	805

	H	-0.2923	-2.1993	0.8344		834	853	888
	H	-1.7344	-1.9633	1.4494		985	1040	1075
	O	-2.7940	0.4719	-0.9746		1107	1191	1645
	H	-3.5466	-0.1199	-0.8703		1656	1658	1687
	H	-2.5646	0.4989	-1.9494		1690	1705	1755
	O	1.3898	2.1262	1.8897		2982	3027	3118
	H	1.5870	1.3519	1.2926		3215	3251	3351
	H	1.9988	2.8289	1.6400		3857	3858	3862
	O	-1.7558	0.4624	-3.3990		3867	3871	3877
	H	-0.8393	0.4557	-3.0216				
	H	-1.7865	1.1799	-4.0397				
$FeCO_3$ –	Fe	0.4584	0.7055	0.7053	0.5582	32	40	41
$(H_2O)_7$	O	0.4957	0.4273	-1.4020	0.5253	57	61	63
	C	-0.3134	-0.6208	-1.2923	0.4700	78	84	92
	O	-0.8411	-1.2282	-2.1969		118	126	136
	O	-0.4654	-0.9396	0.0240		174	203	209
	O	3.0889	-0.7276	-0.7665		226	254	258
	H	2.4064	-0.3515	-1.3547		270	283	291
	H	2.7275	-1.6131	-0.5354		306	316	320
	O	-0.0332	2.7517	0.4840		331	341	354
	H	-0.1840	2.9724	-0.4832		369	390	408
	H	-0.7270	3.1602	1.0119		428	477	514
	O	2.4749	0.7382	1.2482		530	610	623
	H	2.8898	0.1807	0.4997		661	682	699
	H	2.9762	1.5556	1.3412		730	739	775
	O	-0.4272	0.4990	2.7018		788	850	917
	H	0.1361	0.0930	3.3714		978	997	1023
	H	-1.2005	-0.1186	2.5796		1141	1188	1641
	O	-0.1616	2.8647	-2.1244		1648	1654	1660
	H	0.0504	1.8918	-2.1280		1683	1692	1718
	H	-0.9133	2.9892	-2.7125		1755	2783	3073
	O	-2.2468	-1.2841	1.9559		3206	3268	3386
	H	-1.8022	-1.3451	1.0748		3474	3545	3640
	H	-3.1921	-1.2035	1.7954		3843	3858	3866
	O	1.5518	-2.9130	0.0283		3867	3875	3880
	H	0.7183	-2.4009	0.0912				
	H	1.3606	-3.6404	-0.5738				
$FeCO_3$ –	Fe	0.5787	0.5564	0.8431	0.51018	21	35	39
$(H_2O)_8$	O	0.2684	0.6059	-1.2623	0.4149	52	54	62
	C	-0.3633	-0.5493	-1.1790	0.3813	67	84	94
	O	-1.1653	-0.9866	-1.9980		96	106	127
	O	-0.0617	-1.1731	-0.0271		134	159	177
	O	3.1702	-0.0285	-1.1731		191	205	216
	H	2.3515	0.2690	-1.6097		223	243	257
	H	3.0406	-1.0005	-1.0781		271	284	289
	O	-0.2955	2.4756	1.0545		315	328	335

	H	-0.6630	2.8025	0.1823		342	364	376
	H	-0.9848	2.5410	1.7254		390	402	425
	O	2.5893	0.8974	1.1406		442	449	503
	H	2.9883	0.5758	0.2542		508	514	600
	H	2.9394	1.7713	1.3465		618	653	666
	O	-0.4505	0.0616	2.7182		687	693	723
	H	0.0313	-0.3582	3.4400		741	754	765
	H	-1.1754	-0.5922	2.4512		829	866	922
	O	-0.9744	2.9426	-1.4433		1003	1015	1111
	H	-0.5867	2.0548	-1.6564		1154	1238	1633
	H	-1.8636	2.9471	-1.8128		1650	1660	1668
	O	-2.0595	-1.7462	1.7331		1680	1693	1701
	H	-1.3802	-1.8828	1.0383		1716	1737	2747
	H	-2.8419	-1.5245	1.1917		2945	3119	3332
	O	-3.5677	-1.1584	-0.5862		3446	3461	3509
	H	-2.7829	-1.1843	-1.1787		3543	3632	3677
	H	-4.2292	-1.7187	-1.0039		3848	3849	3855
	O	2.2719	-2.5723	-0.6103		3869	3874	3879
	H	1.3648	-2.2694	-0.3874				
	H	2.1681	-3.3125	-1.2172				
$FeCO_3$ – $(H_2O)_9$	Fe	0.7141	0.6248	0.7583	0.4950	31	36	42
	O	0.7576	0.1602	-1.3695	0.3287	48	60	62
	C	-0.1126	-0.7811	-1.1348	0.3080	71	76	86
	O	-0.8514	-1.3127	-1.9682		93	102	107
	O	-0.1725	-1.0931	0.1781		123	130	136
	O	3.3731	-0.8673	-0.6608		164	180	189
	H	2.6643	-0.5596	-1.2579		206	219	235
	H	3.0494	-1.7427	-0.3547		245	255	258
	O	0.1552	2.5988	0.4201		262	269	295
	H	-0.1701	2.7047	-0.5461		304	314	316
	H	-0.4885	3.0033	1.0122		341	369	382
	O	2.7259	0.7049	1.2602		391	406	420
	H	3.1521	0.1142	0.5444		430	443	464
	H	3.2109	1.5360	1.3095		505	520	539
	O	-0.4497	0.6288	2.6251		606	625	635
	H	-0.0995	0.1775	3.4019		653	664	687
	H	-1.3004	0.1572	2.3909		725	736	743
	O	-0.5511	2.5368	-2.0374		772	798	868
	H	-0.0899	1.6864	-2.1885		890	903	922
	H	-1.5093	2.3361	-2.1314		1006	1045	1101
	O	-2.4124	-0.8892	1.6741		1152	1267	1625
	H	-1.7247	-1.2349	1.0609		1653	1663	1670
	H	-3.1217	-0.6237	1.0709		1684	1687	1702
	O	-3.3596	-0.7638	-1.2321		1711	1719	1726
	H	-2.4692	-1.0995	-1.5289		2706	2785	3152
	H	-4.0058	-1.3978	-1.5602		3210	3450	3473

	O	1.8874	-2.9905	0.3722		3500	3506	3548
	H	1.0428	-2.4930	0.3598		3576	3640	3793
	H	1.7058	-3.8353	-0.0525		3848	3853	3858
	O	-3.2505	1.8823	-2.0901		3868	3874	3874
	H	-3.3757	0.9413	-1.8350				
	H	-3.7096	1.9931	-2.9290				
$FeCO_3$ – $(H_2O)_{10}$	Fe	-0.4308	1.3645	0.4613	0.3691	30	31	46
	O	-0.5241	-0.7261	1.0673	0.3250	51	59	64
	C	-0.3069	-1.0732	-0.1695	0.2728	70	74	80
	O	-0.3144	-2.2494	-0.5851		88	89	96
	O	-0.0724	-0.0387	-0.9669		117	119	137
	O	-3.2303	-0.7268	1.5456		143	154	159
	H	-2.2884	-0.9600	1.6803		167	174	181
	H	-3.4834	-1.3517	0.8354		187	205	214
	O	0.7893	1.9089	2.0636		227	228	240
	H	1.3105	1.0817	2.3395		247	252	266
	H	1.3987	2.6519	1.9997		289	298	311
	O	-2.4500	1.6224	0.8362		324	330	335
	H	-2.9056	0.7545	1.1129		379	386	398
	H	-2.9562	2.3786	1.1468		406	414	448
	O	0.2158	2.8946	-0.8939		467	487	502
	H	-0.2625	2.7161	-1.7344		531	576	612
	H	1.1435	2.6299	-1.0783		621	625	649
	O	1.8592	-0.3985	2.5250		663	686	695
	H	1.0503	-0.8460	2.2054		708	733	761
	H	2.5346	-0.6038	1.8459		815	829	851
	O	2.4196	1.2679	-1.3517		868	888	906
	H	1.7118	0.6070	-1.4620		968	1048	1058
	H	3.0693	0.8091	-0.7944		1104	1308	1588
	O	2.2973	-2.6903	-1.2229		1646	1654	1658
	H	1.3236	-2.6387	-1.0587		1667	1687	1692
	H	2.4015	-2.6092	-2.1768		1705	1726	1729
	O	-3.1060	-2.7037	-0.4749		1736	2811	2872
	H	-2.1288	-2.7181	-0.5558		3254	3384	3479
	H	-3.3670	-3.6135	-0.3002		3515	3519	3556
	O	3.5811	-0.8663	0.3087		3570	3584	3593
	H	3.1565	-1.5662	-0.2553		3608	3647	3733
	H	4.4779	-1.1738	0.4799		3860	3863	3869
	O	-0.9623	1.5902	-3.0063		3877	3879	3882
	H	-1.8513	1.5317	-3.3702				
	H	-0.8038	0.7596	-2.5148				

Table S7. Gibbs free energy change (kJ mol^{-1}) for the formation of the *n*th CO_2 cluster of $MgCO_3$ by addition of a single CO_2 molecule to the (*n*-1)th cluster, as a function of temperature (at the B3LYP/6-311+g(2d,p) level of theory). Clusters which are unstable between 70 and 90 km are shown in red typescript.

n	80 K	90 K	100 K	120 K	140 K	150 K
1	-58.81	-57.66	-56.50	-54.19	-51.86	-50.71
2	-44.65	-43.46	-42.27	-39.92	-37.61	-36.46
3	-34.75	-33.36	-31.96	-29.18	-26.42	-25.05
4	-10.84	-9.57	-8.31	-5.81	-3.34	-2.11

Table S8. Gibbs free energy change (kJ mol^{-1}) for the formation of the *n*th H_2O cluster of $MgCO_3$ by addition of a single H_2O molecule to the (*n*-1)th cluster, as a function of temperature (at the B3LYP/6-311+g(2d,p) level of theory). Clusters which are unstable between 70 and 90 km are shown in red typescript.

n	80 K	90 K	100 K	120 K	140 K	150 K
1	-124.45	-123.39	-122.31	-120.13	-117.93	-116.83
2	-99.09	-97.74	-96.36	-93.55	-90.67	-89.21
3	-81.10	-79.74	-78.36	-75.55	-72.70	-71.26
4	-75.74	-74.43	-73.11	-70.40	-67.64	-66.25
5	-64.42	-63.11	-61.77	-59.05	-56.27	-54.87
6	-63.11	-61.84	-60.54	-57.90	-55.20	-53.85
7	-29.16	-27.89	-26.60	-23.98	-21.33	-19.99
8	-33.02	-31.74	-30.44	-27.79	-25.10	-23.75
9	-33.75	-32.38	-30.98	-28.14	-25.25	-23.78
10	-34.35	-33.03	-31.71	-29.04	-26.36	-25.02

Table S9. Gibbs free energy change (kJ mol^{-1}) for the formation of the *n*th CO_2 cluster of $FeCO_3$by addition of a single CO_2 molecule to the (*n*-1)th cluster, as a function of temperature (at the B3LYP/6-311+g(2d,p) level of theory). Clusters which are unstable between 70 and 90 km are shown in red typescript.

n	80 K	90 K	100 K	120 K	140 K	150 K
1	-46.41	-45.06	-43.69	-40.92	-38.13	-36.73
2	-16.94	-16.12	-15.32	-13.78	-12.33	-11.63
3	-8.70	-7.42	-6.16	-3.65	-1.17	0.05
4	-4.78	-3.55	-2.34	0.07	2.45	3.62

Table S10. Gibbs free energy change (kJ mol^{-1}) for the formation of the *n*th H_2O cluster of $FeCO_3$ by addition of a single H_2O molecule to the (*n*-1)th cluster, as a function of temperature (at the B3LYP/6-311+g(2d,p) level of theory). Clusters which are unstable between 70 and 90 km are shown in red typescript.

n	80 K	90 K	100 K	120 K	140 K	150 K
1	-107.34	-106.21	-105.07	-102.75	-100.41	-99.24
2	-64.11	-62.77	-61.41	-58.61	-55.74	-54.30
3	-56.37	-55.19	-54.00	-51.59	-49.15	-47.93
4	-64.83	-63.44	-62.02	-59.13	-56.17	-54.67
5	-32.48	-31.18	-29.86	-27.20	-24.50	-23.14
6	-42.01	-40.70	-39.36	-36.62	-33.82	-32.40
7	-36.16	-34.92	-33.67	-31.15	-28.60	-27.33
8	-29.88	-28.62	-27.33	-24.70	-22.03	-20.68
9	-34.67	-33.28	-31.87	-29.02	-26.11	-24.64
10	-35.30	-33.99	-32.68	-30.02	-27.36	-26.03

Table S11. Critical radius of H_2O-metal carbonate particles for the nucleation and growth of CO_2 ice, as a function of temperature and altitude in the Mars atmosphere.

Altitude / km	95 K	92.5 K	90 K	87.5 K	85 K	82.5 K	80 K
65	1.30	1.01	0.81	0.66	0.55	0.46	0.38
70	1.64	1.23	0.96	0.77	0.63	0.52	0.44
75	2.16	1.52	1.14	0.90	0.73	0.60	0.49
80	3.03	1.94	1.38	1.06	0.84	0.68	0.56
85	4.92	2.63	1.73	1.26	0.97	0.77	0.63
90	12.52	4.02	2.31	1.57	1.17	0.91	0.73
95	-	8.16	3.34	2.03	1.42	1.07	0.84

Movie S1. Trajectory calculation of Mg colliding with CO_2 at a relative velocity of 11.9 km s^{-1}, and producing excited Mg(^{3}P), O(^{3}P) and CO. The total length of the trajectory is 139 fs. The change in potential energy with time is shown in Figure S4.

Movie S2. Trajectory calculation of Mg colliding with CO_2 at a relative velocity of 10.8 km s^{-1}, and producing MgO + CO. The total length of the trajectory is 300 fs. The change in potential energy with time is shown in Figure 3 in the main paper.

References

Forget, F., F. Hourdin, R. Fournier, C. Hourdin, O. Talagrand, M. Collins, S. R. Lewis, P. L. Read, and J.-P. Huot (1999), Improved general circulation models of the Martian atmosphere from the surface to above 80 km, *J. Geophys. Res.*, *104*, 24155-24176, doi: 10.1029/1999JE001025.

Whalley, C. L., and J. M. C. Plane (2010), Meteoric ion layers in the Martian atmosphere, *Faraday Disc.*, *147*, 349-368, doi: 10.1039/c003726e.
