# Supplementary figures and images for: Meteoric Metal Chemistry in the Martian Atmosphere
Source: J Geophys Res Planets. 2018 Mar 6;123(3):695–707. doi: 10.1002/2017JE005510 (PMC5947882; doi:10.1002/2017JE005510)

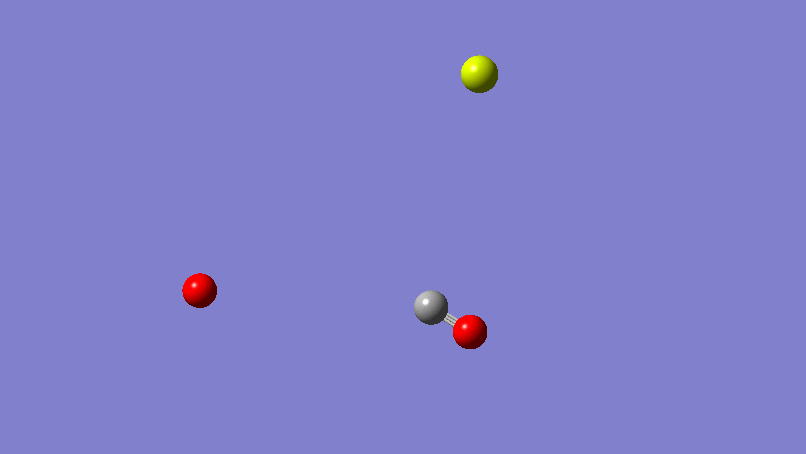

Supplement: Supplementary file 2 — Movie S1 [file JGRE-123-695-s002.gif]

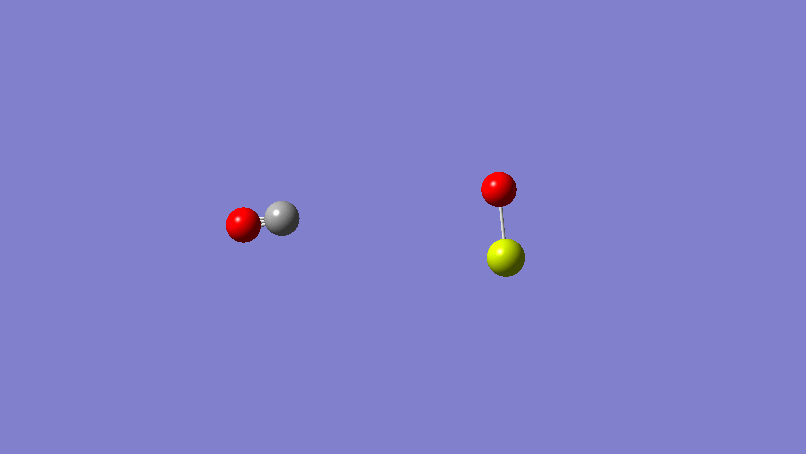

Supplement: Supplementary file 3 — Movie S2 [file JGRE-123-695-s003.gif]
